# Evasion of MAIT cell recognition by the African *Salmonella* Typhimurium ST313 pathovar that causes invasive disease

**DOI:** 10.1073/pnas.2007472117

**Published:** 2020-08-25

**Authors:** Lorena Preciado-Llanes, Anna Aulicino, Rocío Canals, Patrick J. Moynihan, Xiaojun Zhu, Ndaru Jambo, Tonney S. Nyirenda, Innocent Kadwala, Ana Sousa Gerós, Siân V. Owen, Kondwani C. Jambo, Benjamin Kumwenda, Natacha Veerapen, Gurdyal S. Besra, Melita A. Gordon, Jay C. D. Hinton, Giorgio Napolitani, Mariolina Salio, Alison Simmons

**Affiliations:** ^a^Medical Research Council Human Immunology Unit, Medical Research Council Weatherall Institute of Molecular Medicine, University of Oxford, OX3 9DS Oxford, United Kingdom;; ^b^Institute of Integrative Biology, University of Liverpool, L69 7ZB Liverpool, United Kingdom;; ^c^Institute of Microbiology and Infection, School of Biosciences, University of Birmingham, Edgbaston, B11 2TT Birmingham, United Kingdom;; ^d^Malawi–Liverpool–Wellcome Trust Clinical Research Programme, University of Malawi College of Medicine, Blantyre 3, Malawi, Central Africa;; ^e^Department of Biomedical Informatics, Harvard Medical School, Boston, MA 02115;; ^f^Department of Clinical Sciences, Liverpool School of Tropical Medicine, L3 5QA Liverpool, United Kingdom;; ^g^Institute of Infection and Global Health, University of Liverpool, L6 1LY Liverpool, United Kingdom

**Keywords:** *Salmonella* Typhimurium, sequence type 313, ST313, invasive nontyphoidal *Salmonella*, MR1

## Abstract

Mucosal-associated invariant T (MAIT) cells are innate T lymphocytes activated by bacteria that produce vitamin B2 metabolites. Mouse models of infection have demonstrated a role for MAIT cells in antimicrobial defense. However, proposed protective roles of MAIT cells in human infections remain unproven and clinical conditions associated with selective absence of MAIT cells have not been identified. We report that typhoidal and nontyphoidal *Salmonella enterica* strains activate MAIT cells. However, *S.* Typhimurium sequence type 313 (ST313) lineage 2 strains, which are responsible for the burden of multidrug-resistant nontyphoidal invasive disease in Africa, escape MAIT cell recognition through overexpression of *ribB*. This bacterial gene encodes the 4-dihydroxy-2-butanone-4-phosphate synthase enzyme of the riboflavin biosynthetic pathway. The MAIT cell-specific phenotype did not extend to other innate lymphocytes. We propose that *ribB* overexpression is an evolved trait that facilitates evasion from immune recognition by MAIT cells and contributes to the invasive pathogenesis of *S.* Typhimurium ST313 lineage 2.

## Significance

Nontyphoidal *Salmonella* serotypes are a common cause of self-limiting diarrhoeal illnesses in healthy adults. However, recently, a highly invasive multidrug resistant *Salmonella* Typhimurium sequence type 313 has emerged as a major cause of morbidity and mortality in sub-Saharan Africa, particularly in children and immunosuppressed individuals. In this paper we describe escape from MAIT cell recognition as an additional mechanism of immune evasion of *S.* Typhimurium ST313. As MAIT cells represent an early defense mechanism against pathogens at mucosal surfaces, and their frequency and function are altered in immunosuppressed individuals in sub-Saharan Africa, harnessing their function may offer an important therapeutic strategy to improve mucosal immunity.

The gram-negative bacterium *Salmonella enterica* spp. comprises many serovars which are closely related phylogenetically but cause very different disease presentations and distinct immune responses in immunocompetent hosts (1, 2). Infection by the human restricted *Salmonella* typhoidal serovars (*S.* Typhi and *S.* Paratyphi) results in a severe systemic disease called enteric fever. In contrast, nontyphoidal serovars originating from zoonotic reservoirs such as *S.* Typhimurium and *S.* Enteritidis, cause self-limiting diarrheal disease in healthy individuals (1–3). Multidrug resistant *S.* Typhimurium strains of a distinct multilocus sequence type 313 (ST313) recently emerged in sub-Saharan Africa. *S.* Typhimurium ST313 is associated with invasive blood stream infections in immunocompromised individuals and is distinct from the *S.* Typhimurium strains that cause gastroenteritis globally.

Since it was first reported in 2009 (4), the *S.* Typhimurium ST313 clade has become the major cause of invasive nontyphoidal *Salmonella* (iNTS) disease in Africa (5, 6) and comprises two subclade lineages (6), termed lineages 1 and 2. Bacteraemia by iNTS causes an estimated 77,500 deaths annually worldwide (7), primarily in Africa, among young children with recent malaria, malarial anemia, or malnutrition and in adults afflicted with HIV, among whom recurrent disease is also common (1, 5, 8–11). *S.* Typhimurium ST313 isolates have rarely been reported outside of Africa (4) and African ST313 blood isolates are genetically distinct from rare diarrheal ST313 isolates found in the United Kingdom (12) or Brazil (13). Genotypic and phenotypic analyses of several clinical isolates of the two well-described ST313 lineages identified signatures of metabolic adaptation and unique enteropathogenesis in animal models, consistent with adaptation to invasive disease in an immunocompromised human population (4, 14, 15).

B and T cell responses can mediate a protective role in mouse models of *Salmonella* infection. B cells provide the first line of defense at mucosal sites to restrain systemic dissemination, while T cells are needed for *Salmonella* clearance (16–18). Cross-reactive and serovar-specific MHC-restricted T cell responses have been well characterized in humans (19–24). *Salmonella* can also induce activation of non-MHC-restricted T cells, specifically γδ T cells, invariant natural killer T cells (iNKTs), and mucosal-associated invariant T (MAIT) cells (25–27), although their protective role remains undetermined.

MR1-restricted MAIT cells comprise a highly conserved class of semi-invariant T cells, bridging innate and adaptive immunity (28). The MHC class I-like molecule MR1, bound to derivatives of vitamin B2 intermediates, activates MAIT cells (29). This process can drive antibacterial activity, in vitro and in vivo, and correlates with the presence of the vitamin B2 biosynthetic pathway in several commensal and pathogenic bacteria and fungal species (reviewed in ref. 30). Similar to iNKT and γδ T cells, MAIT can be activated by cytokines (IL-12, IL-18, type I IFN) independently of their TCR engagement (31). The ability of MAIT cells to recognize *S.* Typhimurium-infected targets (32) prompted the identification, within bacterial supernatants, of the potent MAIT cell agonists (lumazine and pyrimidines), derivatives of the vitamin B2 intermediate 5-A-RU (29, 33). Following intranasal infection with *S.* Typhimurium, murine MAIT cells become activated and accumulate in the lungs (26). Human challenge studies with typhoidal serovars (*S.* Typhi and *S.* Paratyphi A) also demonstrated sustained MAIT cell activation and proliferation at peak of infection (34, 35).

While many commensal and pathogenic bacteria possess the riboflavin biosynthetic pathway, the levels of resulting MAIT stimulation varies (36, 37), perhaps reflecting the influence of microenvironment on bacterial metabolism and antigen availability. The ability of MAIT cells to recognize and respond to several isolates of the same pathogen may also vary depending on metabolic differences between isolates (38).

We hypothesized that MAIT cells contributed to the cellular response to *Salmonella enterica* serovars responsible for invasive disease, and examined the ability of MAIT cells to recognize and respond to different *S. enterica* serovars associated with invasive disease in Africa. Here, we demonstrate that *S.* Typhimurium ST313 lineage 2 isolates escape MAIT cell recognition through overexpression of RibB, a bacterial enzyme of the riboflavin biosynthetic pathway. Our results suggest that MAIT cell immune protection represents an important “evolutionary bottleneck” for the pathogen.

## Results

### Identification of Cellular Responses to Multiple *Salmonella enterica* subsp *enterica* Serovars.

To identify potential differences in the response of innate and adaptive T cells to distinct *Salmonella* pathovars, we focused on two pathovariants of *S.* Typhimurium that are responsible for different types of human disease. *S.* Typhimurium ST313 is associated with invasive disease among immunocompromised individuals in Africa and a representative isolate is D23580 (STM-D23580). *S.* Typhimurium sequence type 19 (ST19) causes noninvasive diarrheal infections in immunocompetent individuals globally (a representative isolate is LT2, designated STM-LT2). Peripheral blood mononuclear cells (PBMCs) isolated from healthy donors were infected with both *S.* Typhimurium pathovariants, and *S.* Typhi strain Ty2 (ST-Ty2) was used to represent a more distantly related serovar that causes invasive disease in immunocompetent individuals. *Escherichia coli* (*E. coli*) was included as unrelated control. Upon infection, PBMCs were incubated overnight in the presence of brefeldin A to permit intracellular cytokine accumulation. T lymphocytes were stained with a panel of fluorescently labeled antibodies to simultaneously identify different T cell populations (MAIT, γδ, CD4, and CD8) and determine their activation status (CD69) and cytokine production (IFN-γ and TNF-α).

We first defined the heterogeneity of T cell responses to *Salmonella* by performing an unsupervised clustering analysis on all CD3^+^ T cells expressing the activation marker CD69 following overnight incubation with the *Salmonella* pathovariants. Dimensionality reduction analysis by t-distributed stochastic neighbor embedding (*t*-SNE) revealed 22 populations of CD3^+^ CD69^+^ T cells, some of which differed in frequency according to the infecting *Salmonella* pathogen. Clusters were then annotated and assigned to MAIT (identified as CD3^+^ Vα7.2^+^ CD161^high^), γδ^+^, CD4^+^, or CD8^+^ T cell subsets based on the expression of distinct phenotypic markers.

**Fig. 1.**
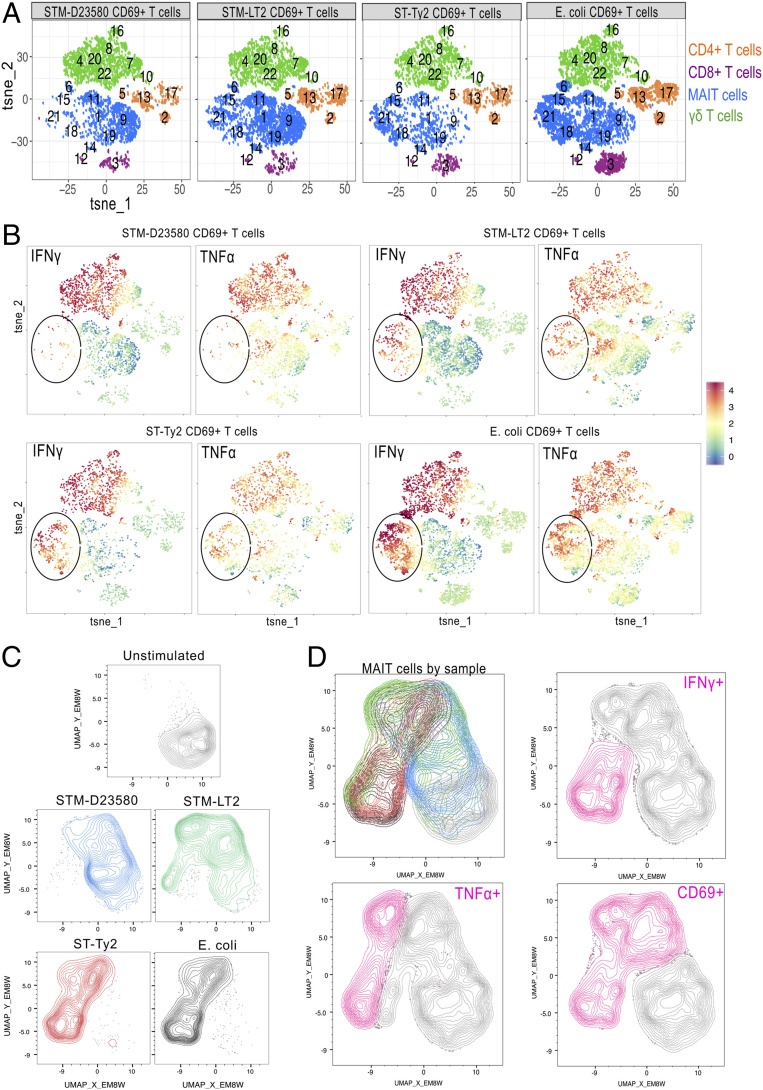
Identification of cellular responses to multiple *S. enterica* spp. *enterica* serovars. PBMCs were left unstimulated or infected at MOI of 5 with STM-D23580, STM-LT2, ST-Ty2, or *E. coli*. Intracellular staining was performed to detect CD69 expression and cytokine production (TNF-α and IFN-γ), as correlates of T cell activation. (*A*) *t*-SNE plots on gated CD69^+^ CD3^+^ T cells infected with STM-D23580, STM-LT2, ST-Ty2, or *E. coli*. Four CD3^+^ T cell populations (CD4^+^, CD8^+^, γδ^+^, and MAIT) were annotated based on the expression of distinct phenotypic markers. CD4, CD8, TCRγδ, Vα7.2^+^, CD161, IFN-γ, and TNF-α were the parameters included for *t*-SNE analysis. Plots correspond to one representative donor. (*B*) *t*-SNE plots as in *A* showing relative expression of TNF-α and IFN-γ on CD69^+^ CD3^+^ T cells. (*C*) UMAP analysis on concatenated CD3^+^ Vα7.2^+^ CD161^+^ MAIT cells from the same donor as in *A* and *B*. Calculated UMAPs are shown for each experimental condition. CD69, IFN-γ, and TNF-α were the parameters included for analysis. (*D*, *Top Left*) UMAP as an overlay of concatenated MAIT cell populations from *C*: unstimulated in light gray, STM-D23580 in blue, STM-LT2 in green, ST-Ty2 in red, and *E. coli* in dark gray. (*D*, *Top Right* and *Bottom*) UMAPs showing expression of CD69, TNF-α, and IFN-γ in pink. (*A*–*D*) Data from one donor representative of four biological replicates.

We discovered that a group of clusters of MAIT cells (clusters 6, 15, 18, and 21) was underrepresented among all CD69^+^ cells upon infection with STM-D23580, compared with STM-LT2, ST-Ty2, and *E. coli* (Fig. 1*A*). Next, we analyzed expression of IFN-γ and TNF-α in CD69^+^ activated T cells. We determined that the underrepresented clusters of CD69^+^ MAIT cells represented IFN-γ- and TNF-α-producing MAIT cells (Fig. 1*B*).

MAIT cells were next analyzed using uniform manifold approximation and projection (UMAP), a neighboring dimensionality reduction technique that preserves embedding and global distances better than *t*-SNE (39), and clearly defined the trajectory of the distinct subpopulations of *Salmonella*-activated MAIT cells (Fig. 1*C*). STM-LT2-stimulated MAIT cells clustered close to MAIT cells stimulated with ST-Ty2 and *E. coli*, which were characterized by elevated expression of CD69 and the presence of single and double producers of TNF-α and IFN-γ. In contrast, STM-D23580-stimulated MAIT cells clustered closer to unstimulated cells, away from MAIT cells stimulated with ST-Ty2 and *E. coli* (Fig. 1 *D*, *Top Left*). STM-D23580-stimulated MAIT cells expressed low levels of CD69, with only a small TNF-α-producing subpopulation and almost no IFN-γ producing cells (Fig. 1*D*).

### *S.* Typhimurium ST313 Lineage 2 Fails to Elicit MAIT Cell Activation in Healthy and Susceptible Individuals.

**Fig. 2.**
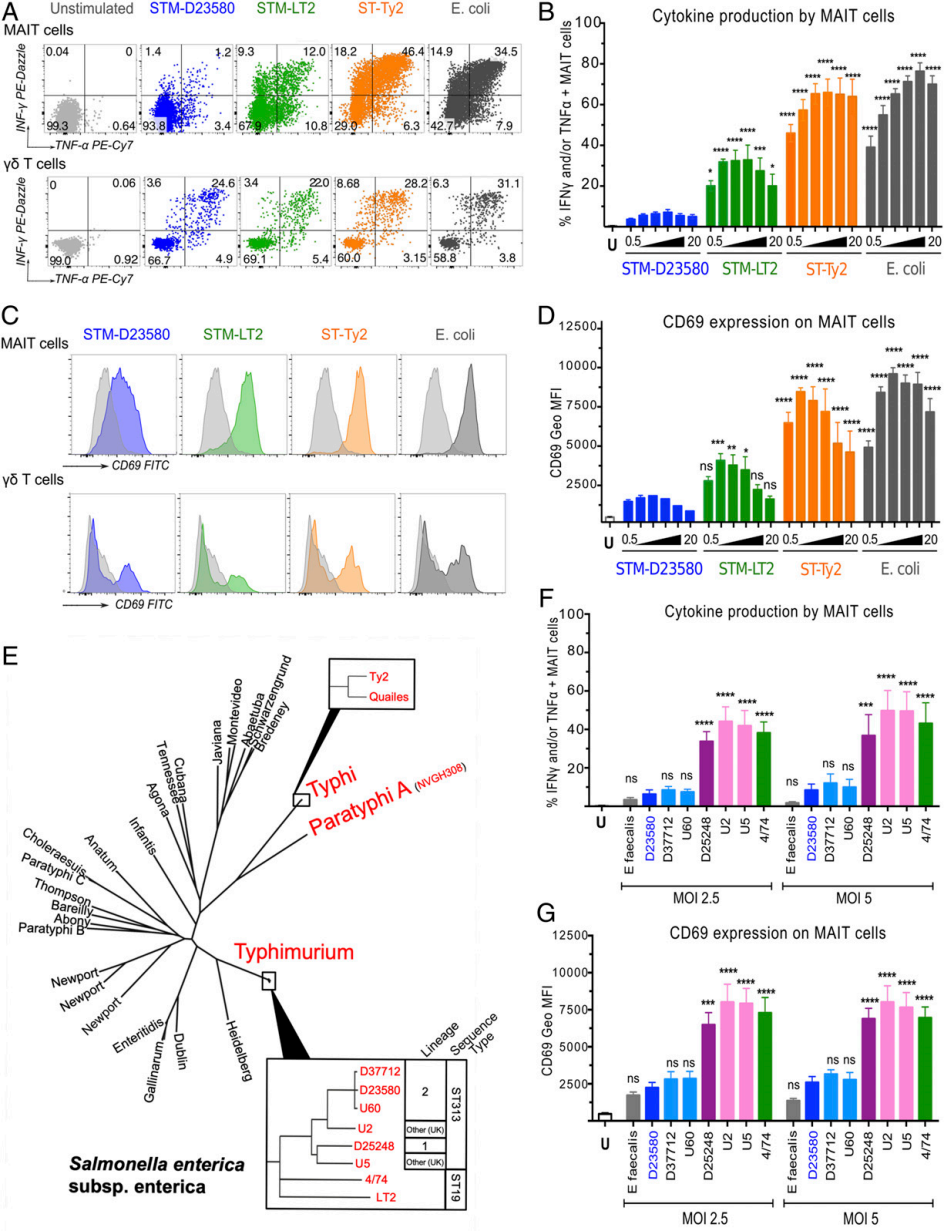
*S.* Typhimurium ST313 lineage 2 fails to elicit MAIT cell activation. PBMCs were left unstimulated (U) or infected with a variety of *Salmonella* strains at the indicated MOI. *E. coli* was included as positive control and *E. faecalis* as negative control. (*A*) Production of TNF-α and IFN-γ by MAIT and γδ^+^ T cells was detected by intracellular staining. Representative flow cytometry plots from one volunteer are shown. (*B*) Percentage of TNF-α and/or IFN-γ producing MAIT cells when stimulated at increasing MOI, from 0.5 to 20 bacteria per cell. Data are represented as mean ± SEM, two-way ANOVA + Dunnet’s, *n =* 4. (*C*) CD69 staining profile of stimulated MAIT and γδ^+^ T cells. Representative histograms from one volunteer are shown. (*D*) CD69 expression on MAIT cells when stimulated as in *B*. Data are represented as geometric mean ± SEM, two-way ANOVA + Dunnet’s, *n =* 4. (*E*) Phylogenetic relationships between strains used in these experiments (red) within the context of *S. enterica* phylogeny. (*F*) Percentage of TNF-α- and/or IFN-γ-producing MAIT cells, treated with bacterial strains at MOI of 2.5 and 5. Data are represented as mean ± SEM, two-way ANOVA + Dunnet’s, *n =* 4. (*G*) Levels of CD69 expression on MAIT cells treated as in *E*. Data are represented as geometric mean ± SEM, two-way ANOVA + Dunnet’s, *n =* 4.

To validate our unsupervised analysis, we infected PBMCs with the different *Salmonella* strains at increasing multiplicity of infection (MOI) and then assessed MAIT cell activation by flow cytometry. Infection by STM-D23580 consistently induced limited MAIT cell responses across a range of MOIs and in every healthy donor tested. In comparison with STM-LT2, ST-Ty2, or *E. coli*, STM-D23580-stimulated MAIT cells significantly expressed less CD69 and produced less IFN-γ and TNF-α (Fig. 2 *A*–*D*). This effect was not dependent on a selective loss of MAIT cells, as STM-D23580 did not have a detrimental effect on MAIT cell viability (*SI Appendix*, Fig. S1*A*). γδ T cells, a subset of innate T lymphocytes also present in PBMCs, were strongly activated by STM-D23580, indicating that the lack of activation is a phenomenon limited to MAIT cells (Fig. 2 *A* and *C*).

In culture, *Salmonella* spp. secrete vitamin B2 intermediates that can bind to MR1 on antigen-presenting cells (APCs), to trigger MAIT cell activation (29). To examine whether STM-D23580 secretes MAIT cell agonists, we collected supernatants from single-colony cultures to stimulate PBMCs. Supernatants from STM-LT2 and *E. coli* induced a dose-dependent production of IFN-γ and TNF-α by MAIT cells, whereas STM-D23580 supernatants did not (*SI Appendix*, Fig. S1*B*).

To validate such findings, we assessed MAIT cell responses to a broader selection of bacterial isolates, including two *S*. Typhi strains (ST-Ty2 and ST-Quailes) and one *S.* Paratyphi A strain; in addition two differently sourced stocks of STM-D23580 were tested, to ensure that genuine sequence type 313 isolates were being used. At two different MOIs, STM-D23580 elicited the lowest levels of MAIT cell activation of the group (*SI Appendix*, Fig. S1 *C* and *D*). In contrast, γδ T cell responses were comparable across all *Salmonella* pathovars (*SI Appendix*, Fig. S1 *E* and *F*).

We next determined whether the lack of MAIT cell activation was caused by the entire *S.* Typhimurium ST313 clade or was a unique characteristic of ST313 lineage 2 which is currently causing most clinical disease in Africa (14). STM-D23580 and additional isolates of ST313 lineage 2, were compared with closely related isolates that were members of ST313 lineage 1 or the UK ST313 group that is associated with gastroenteritis (Fig. 2*E*). To examine MR1-independent MAIT cell activation, we used *Enterococcus faecalis* as a negative control as it lacks the vitamin B2 biosynthetic pathway (29).

Remarkably, only the ST313 strains belonging to lineage 2, such as D23580, D37712, and U60, failed to elicit MAIT cell activation (Fig. 2 *F* and *G*). All other *Salmonella* ST313 lineages tested (strains U2, U5, and D25248) triggered the same level of MAIT cell responses as *S*. Typhimurium 4/74 (STM-4/74) (40), a sequence type 19 strain that is closely related to STM-LT2 and is associated with noninvasive diarrheal infections.

**Fig. 3.**
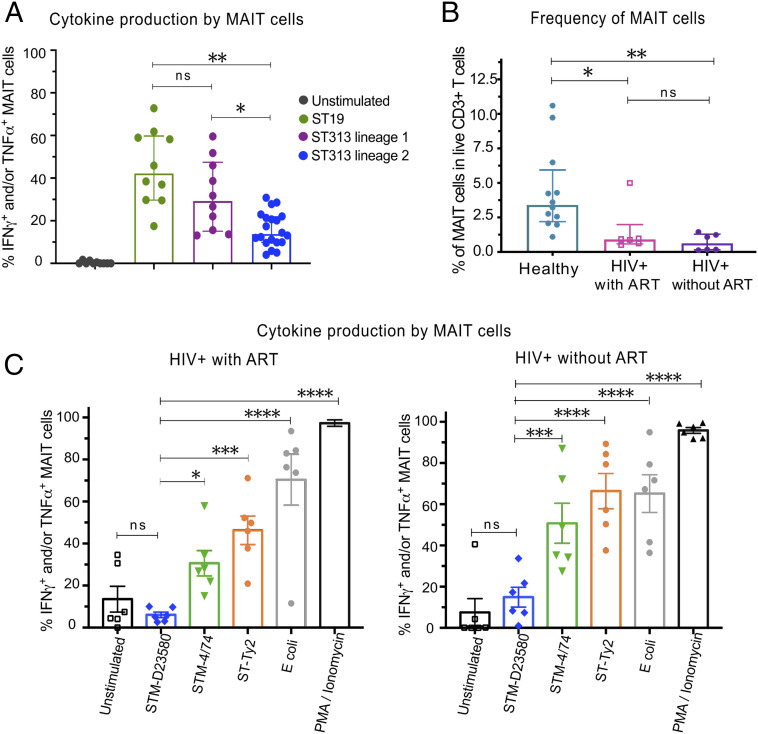
Characterization of MAIT cell responses to *Salmonella* spp. in relevant and susceptible cohorts of individuals. (*A*) PBMCs were isolated from 10 healthy individuals living in Malawi. Cells were infected at MOI of 7 with various strains from sequence type 313 (ST313) lineages 1 and 2 or with the sequence type 19 (ST19) reference strain STM-4/74. Each dot represents the average of TNF-α- and/or IFN-γ-producing MAIT cells per individual upon stimulation with four strains from lineage 1 and eight strains from lineage 2 (*SI Appendix*, Fig. S2). Median ± interquartile range (IQR), Friedman + Dunn’s, *n =* 10 for each group. (*B*) Percentage of MAIT cells (CD3^+^ Vα7.2^+^ CD161^+^) in PBMCs isolated from healthy (*n =* 12) and HIV-infected individuals living in Malawi, with (*n =* 6) or without ART (*n =* 6). Data are represented as percentage of live CD3^+^ T cells, median with IQR, Kruskal–Wallis + Dunn’s. (*C*) PBMCs isolated from HIV^+^ patients with or without ART were infected at MOI of 7 with either STM-D23580, STM-4/74, STy-H58, or *E. coli*. Phorbol 12-myristate 13-acetate (PMA)/ionomycin was used as positive control. Data are represented as percentage of TNF-α- and/or IFN-γ-producing MAIT cells, mean ± SEM, one-way ANOVA + Dunnet’s, *n =* 6 for each group. ns = non-significant.

To confirm our observations in a relevant population, we performed a series of assays on blood samples obtained from healthy adult residents of Malawi, an endemic area for iNTS infections caused by ST313 strains. We expanded our investigation by using additional ST313 isolates and infected PBMCs with four strains from lineage 1 and eight strains from lineage 2. In comparison to the sequence type 19 representative strain STM-4/74, PBMCs infected with ST313 lineage 1 strains elicited similar MAIT cell responses, whereas infection with ST313 lineage 2 induced significantly lower levels of MAIT cell responses (Fig. 3*A* and *SI Appendix*, Fig. S2).

Lastly, we extended our findings to a clinically susceptible cohort of HIV-infected adults living in Malawi. In comparison with a cohort of healthy samples from the United Kingdom and Malawi, and consistent with previous reports (reviewed in ref. 41), the overall percentage of MAIT cells was reduced among HIV-infected individuals, particularly in those not receiving antiretroviral therapy (ART) (Fig. 3*B*). In line with the data obtained with healthy volunteers, MAIT cells from HIV^+^ adults also failed to produce IFN-γ and TNF-α following ex vivo stimulation with STM-D23580, regardless of their ART status (Fig. 3*C*). In contrast, MAIT cells from HIV^+^ individuals responded strongly to *S.* Typhi and STM-4/74.

These findings suggest that evasion of MAIT cell recognition by sequence type 313 *Salmonella* strains may be a critical factor during the course of natural iNTS disease in endemic populations and in clinically susceptible groups.

### STM-D23580 Does Not Affect MR1-Dependent Antigen Presentation.

**Fig. 4.**
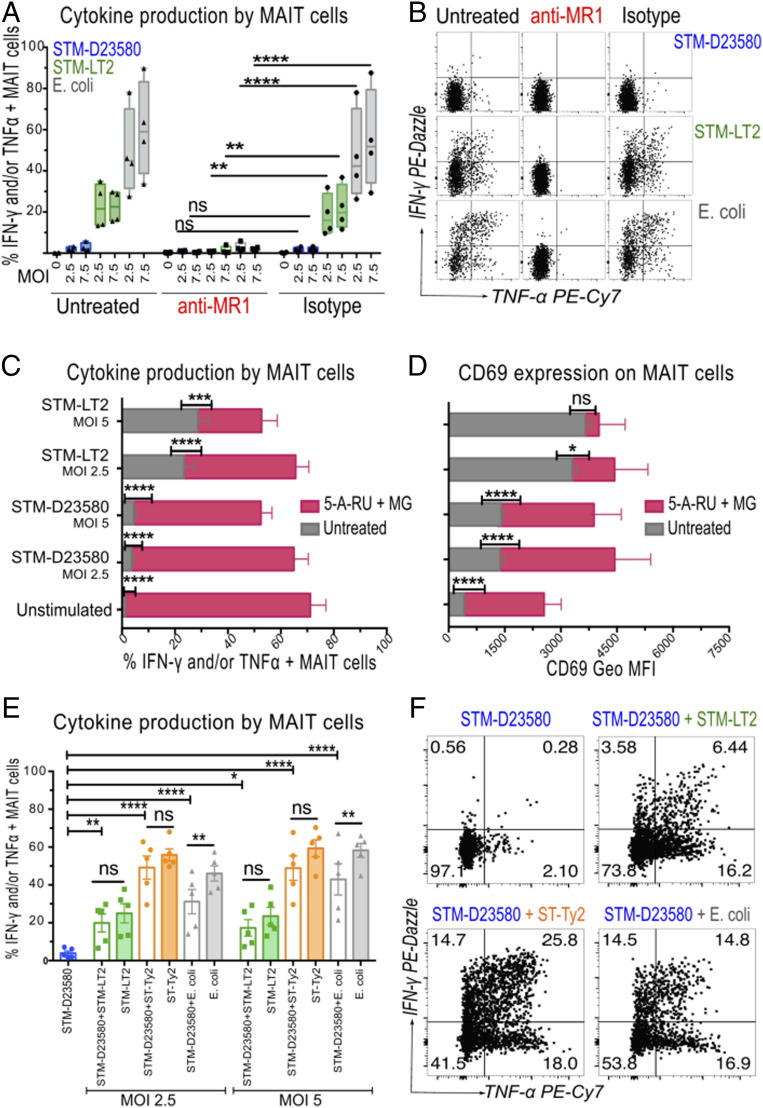
STM-D23580 does not affect MR1-dependent antigen presentation. (*A*) PBMCs were infected (at MOI 2.5 and 7.5) with STM-D23580 (blue), STM-LT2 (green), or *E. coli* (gray), and incubated in the presence of anti-MR1 blocking antibody or the equivalent isotype control. Data are represented as percentage of TNF-α- and/or IFN-γ-producing MAIT cells, box-and-whisker plot, two-way ANOVA + Dunnet’s, *n =* 4. (*B*) Representative example of cytokine production by stimulated MAIT cells treated as in *A*. (*C* and *D*) PBMCs were left unstimulated or infected (at MOI 2.5 and 5) with either STM-D23580 or STM-LT2, in the presence (pink bars) or absence (gray bars) of the MR1 ligands 5-A-RU and MG. Percentage of cytokine-producing MAIT cells and their CD69 expression are shown. Data are represented as mean and geometric mean ± SEM, two-way ANOVA + Bonferroni’s, *n =* 4. (*E*) PBMCs were infected with D23580 at MOI of 2.5, alone or in combination with STM-LT2 (green), ST-Ty2 (orange), or *E. coli* (gray), at two different MOI (2.5 and 5). Data are represented as percentage of TNF-α-and/or IFN-γ-producing MAIT cells, mean ± SEM, one-way ANOVA + Sidak’s, *n =* 5. (*F*) Representative example of cytokine production by stimulated MAIT cells treated as in *E*.

To define the molecular mechanisms underlying the lack of MAIT stimulation by STM-D23580, we first investigated whether MAIT cell activation was MR1 dependent. Adding the anti-MR1 blocking antibody 26.5 (42) completely abrogated the MAIT cell activation induced by STM-LT2 and *E. coli*, as well as the minimal activation induced by STM-D23580 (Fig. 4 *A* and *B*).

We next examined whether STM-D23580 either failed to produce stimulatory MR1 ligands or actively inhibited MAIT cell activation. MAIT cell activation was restored following the addition of the canonical MAIT cell ligand 5-amino-6-D-ribitylaminouracil (5-A-RU) and methylglyoxal (MG) (33) to infected PBMCs (Fig. 4 *C* and *D*), demonstrating that a dominant antagonistic MR1 ligand was not released by STM-D23580. Consistent with these results, a combination of supernatants from overnight cultures of both STM-D23580 and STM-LT2 (added simultaneously or 1 h apart) fully restored MAIT cell activation (*SI Appendix*, Fig. S3 *A* and *B*). Likewise, coinfection of PBMCs with STM-D23580 plus either STM-LT2, ST-Ty2, or *E. coli*, also restored MAIT cell activation to the levels observed with single infections (Fig. 4 *E* and *F*). Taken together, our data show that STM-D23580 neither interferes with nor blocks MAIT cell activation in the presence of stimulatory MR1 ligands.

To exclude the possibility that the lack of MAIT cell activation arose from an insufficient infection of APCs, we exposed monocyte-derived dendritic cells (MoDCs) to fluorescently labeled STM-D23580 or STM-LT2. Live *Salmonella*-containing MoDCs were sorted by fluorescence-activated cell sorting (FACS) and cocultured with enriched autologous CD3^+^ T lymphocytes, as described previously (43). In contrast to STM-LT2-infected MoDCs, STM-D23580-infected MoDCs did not stimulate effector MAIT cells (*SI Appendix*, Fig. S3*C*). Using an MR1-overexpressing antigen-presenting cell line, we found that STM-D23580 supernatants do not cause down-regulation of surface MR1 expression, thus excluding this as a possible cause of the lack of MAIT cell activation (*SI Appendix*, Fig. S3*D*).

Cytokines, such as IL-12, IL-18, and type I IFN, released by APCs upon bacterial or viral infection can also activate MAIT cells in a MR1-independent manner (31). We confirmed that when MoDCs were cocultured with purified MAIT cells, equal amounts of bioactive IL-12p70 were secreted upon infection with STM-D23580, STM-LT2, and *E. coli* (*SI Appendix*, Fig. S3*E*).

Taken together, these observations refuted the hypothesis that lack of MAIT cell activation is caused by an impaired MR1-dependent antigen presentation following STM-D23580 infection.

### STM-D23580 Evades MAIT Cell Recognition by Overexpression of RibB.

**Fig. 5.**
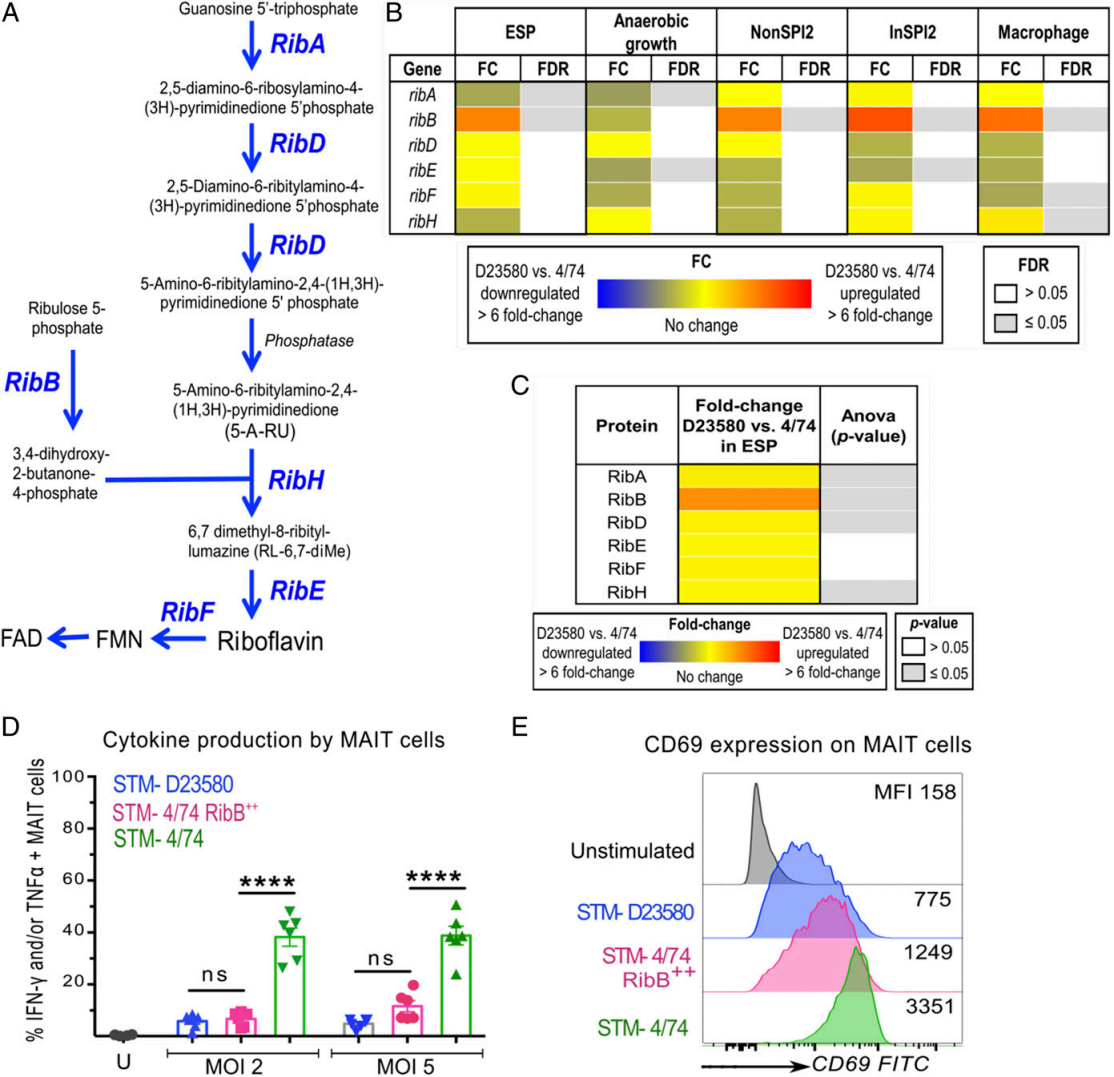
STM-D23580 evades MAIT cell recognition by overexpression of *ribB*. (*A*) Schematic representation of *Salmonella*’s riboflavin pathway, adapted from Soudais et al. (45). (*B*) The relative transcriptional expression levels of the *ribABDEFH* genes were derived from our published RNA-seq dataset (44). The gene expression values from STM-D23580 and STM-4/74 were determined in five infection-relevant conditions: ESP, anaerobic growth, NonSPI2 (SPI2-noninducing PCN), InSPI2 (SPI2-inducing PCN), and macrophage (intra-RAW264.7 murine macrophage environment). Values indicate fold change (FC) and FDR, calculated from a Voom/Limma analysis (using Degust) for the RNA-seq data comparison of STM-D23580 versus STM-4/74. Data represent three biological replicates. (*C*) The relative expression levels of the RibABDEFH proteins were derived from our published proteomic dataset for the ESP condition (44). The heat map shows differential expression analysis following comparison between STM-D23580 and STM-4/74. Results from LC-MS/MS were analyzed using Progenesis QI software (Nonlinear Dynamics) for label-free quantification analysis. Each sample represents six biological replicates. Data represented in *B* and *C* were extracted from Canals et al. (44). (*D*) PBMCs were infected at two different MOI (2 and 5) with STM-D23580 (blue), STM-4/74 (green), or STM-4/74 RibB^++^ (pink). Data are represented as percentage of TNF-α- and/or IFN-γ-producing MAIT cells, mean ± SEM, two-way ANOVA + Tukey’s, *n =* 6. (*E*) CD69 staining profile of stimulated MAIT cells treated as in *D*. Representative histograms from one volunteer are shown. MFI, median fluorescence intensity.

The observation that STM-D23580 and related ST313 isolates do not interfere with MR1 presentation or cytokine production suggests that these bacteria might not produce the MR1 binding ligands that are generated by other *S.* Typhimurium or *S.* Typhi pathovariants. The major source of natural antigens driving MAIT cell activation derives from byproducts of microbial riboflavin synthesis (33). We depict the *Salmonella* riboflavin biosynthetic pathway in Fig. 5*A*. A comparison of the coding sequences (CDSs) of the enzymes involved in the riboflavin biosynthesis pathway found only one nucleotide change (SNP) between the sequence type 19 STM-4/74 and the sequence type 313 STM-D23580 strains. This synonymous coding variant was located in the *ribD* gene (Glu316Glu) (44), suggesting that there were no biochemical differences between the riboflavin biosynthesis pathways of the sequence type 313 and sequence type 19 isolates.

To determine whether the enzymes of the riboflavin pathway of the sequence type 19 STM-4/74 and sequence type 313 STM-D23580 strains were expressed at different levels, we investigated the transcriptomic and proteomic data from our recent comparative analysis (44). Strains STM-4/74 and STM-D23580 are closely related, sharing 92% of coding genes (44). Differential gene expression analysis of the *rib* genes at the transcriptomic level identified significant up-regulation of *ribB* (≥2 fold change, false discovery rate [FDR] ≤ 0.001) in STM-D23580 in four of the five experimental conditions (Fig. 5*B*). We then examined data from a quantitative proteomic approach which showed that RibB protein levels were up-regulated in STM-D23580 compared to STM-4/74, during growth in rich medium at early stationary phase (ESP) (Fig. 5*C*).

We searched for a molecular explanation for the high levels of *ribB* expression in STM-D23580 compared to STM-4/74. In STM-D23580, *ribB* and its 5′ untranslated region (5′ UTR) are transcribed as a single transcript that is initiated from a single gene promoter which we identified previously (44). By analogy with the genetic mechanism identified for overexpression of the PgtE virulence factor in STM-D23580 (46) we searched for nucleotide polymorphisms that distinguished the *ribB* regions of the two strains. There were no differences between the promoter sequences of the *ribB* genes or the 5′ UTR of the strains STM-D23580 and STM-4/74.

To determine whether the MAIT cell activation phenotype was linked to the overexpression of the RibB enzyme (4-dihydroxy-2-butanone 4-phosphate synthase), we created a derivative of STM-4/74 that expressed high levels of RibB. Since deletions in the riboflavin biosynthetic pathway genes are lethal without high dose riboflavin supplementation (33, 47) and *ribB* is essential for *Salmonella* in vivo virulence (48), we used a gene cloning approach to overexpress the *ribB* gene of STM-D23580 in STM-4/74 from a recombinant plasmid (STM-4/74 RibB^++^). The expression of high levels of the RibB enzyme by STM-4/74 ablated MAIT cell activation induced by wild-type STM-4/74. Infection with STM-4/74 RibB^++^ induced low levels of cytokine production and CD69 expression by MAIT cells, recapitulating the phenotype of STM-D23580 (Fig. 5 *D* and *E*). Importantly, γδ T cells responded equally to both STM-4/74 wild type and STM-4/74 RibB^++^ (*SI Appendix*, Fig. S4).

We next evaluated the bacterial growth rate, as well as the infection efficiency of the STM-4/74 RibB^++^ bacterial strain. Midlog phase curves demonstrated no significant differences in the growth rate between STM-4/74 wild type, STM-4/74 RibB^++^, and other related strains (*SI Appendix*, Fig. S5*A*). Human monocyte-derived dendritic cells and human monocyte-derived macrophages were infected in vitro, and the number of intracellular bacterial colony-forming units (cfu) was measured as a readout of internalization. In both cell types tested, the number of recovered intracellular STM-4/74 RibB^++^ was comparable to the number obtained from other *Salmonella* strains (*SI Appendix*, Fig. S5*B*). These data exclude the possibility that the lack of MAIT activation by STM-4/74 RibB^++^ reflects slow growth or poor bacterial internalization into antigen-presenting cells.

**Fig. 6.**
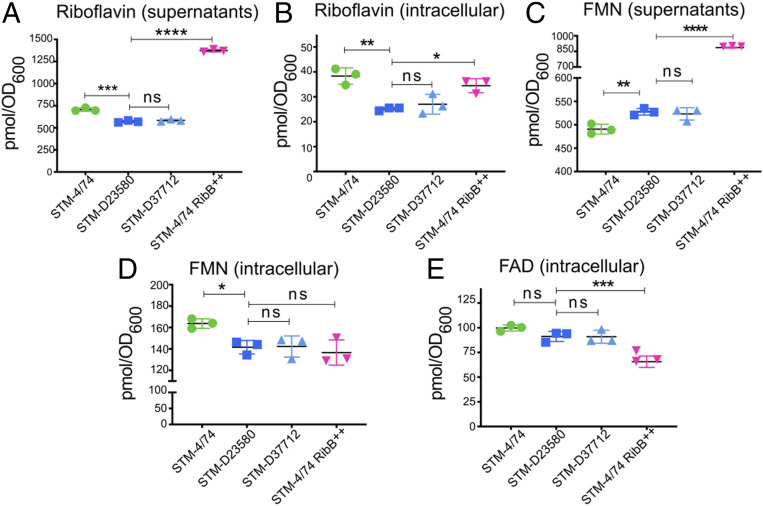
STM-4/74 RibB^++^ has the lowest intracellular levels of FMN, a negative regulator of *ribB* gene expression. Supernatants from early stationary phase and bacterial pellets were harvested and analyzed by HLPC using riboflavin, FMN, and FAD standards. Data are reported in picomole and have been normalized to the absorbance (OD_600_) from each culture. (*A*) Riboflavin levels in supernatants. (*B*) Intracellular riboflavin levels. (*C*) FMN levels in supernatants. (*D*) Intracellular FMN levels. (*E*) Intracellular FAD levels. Measurements were obtained from three biological replicates, mean ± SEM, one-way ANOVA + Dunnet’s.

MAIT activator ligands such as 5-OP-RU (5-[2-oxopropylideneamino]-6-D-ribitylaminouracil) or 5-OE-RU (5-[2-oxoethylideneamino]-6-D-ribitylaminouracil), products of the riboflavin pathway, cannot be measured due to their unstable nature (33). Therefore, to investigate whether overexpression of RibB altered the balance of downstream products from the riboflavin pathway, we measured the amount of riboflavin, flavin mononucleotide (FMN), and flavin adenine dinucleotide (FAD) by high performance liquid chromatography (HPLC). Following growth to early stationary phase, intracellular samples and culture supernatants from STM-4/74 and STM-4/74 RibB^++^ contained larger amounts of riboflavin than the sequence type 313 strains STM-D23580 and STM-D37712 (Fig. 6 *A* and *B*). The STM-4/74 RibB^++^ supernatant with the highest level of riboflavin also contained the largest amount of FMN, compared with STM-4/74, STM-D23580, and STM-D37712 (Fig. 6*C*).

Overall, the data show that the RibB overproducing strain (4/74 RibB^++^) produced the highest level of extracellular riboflavin and FMN, while having the lowest level of intracellular FMN. In contrast, the STM-4/74 wild-type strain had the largest intracellular amount of FMN, compared with 4/74 RibB^++^ and both of the sequence type 313 strains (Fig. 6*D*). While intracellular FAD levels were similar between STM-4/74, STM-D23580, and STM-D37712, STM-4/74 RibB^++^ contained the lowest level of intracellular FAD (Fig. 6*E*). We conclude that the lowest levels of intracellular FMN was found in the STM-D23580, STM-D37712, and STM-4/74 RibB^++^ strains that overexpress the RibB enzyme.

### Reduced MAIT Cell Antibacterial Activity against *S.* Typhimurium ST313 Lineage 2-Infected Macrophages.

To test the role of MAIT cells in clearing *Salmonella* infections, we developed an in vitro assay where human monocyte-derived macrophages were infected with the different *Salmonella* strains, either in presence or absence of purified MAIT cells. As observed with PBMCs, we measured less IFN-γ in supernatants from cocultures of purified MAIT cells and macrophages infected with STM-4/74 RibB^++^ and sequence type 313 lineage 2 strains, as compared to the control sequence type 19 strains (*SI Appendix*, Fig. S6*A*).

In these experiments, the number of intracellular colony-forming units recovered from infected macrophages was used as a surrogate of bactericidal activity induced by MAIT cells. In the majority of the biological replicates and in comparison with macrophages alone, we observed a modest reduction of colony-forming unit numbers (25% or less) when MAIT cells and sequence type 313 lineage 2-infected macrophages were put in coculture (*SI Appendix*, Fig. S6*B*). In contrast, almost all biological replicates infected with sequence type 19 *Salmonella* had a reduction of more than 25% in the number of colony-forming units when MAIT cells were added to the infected macrophages (*SI Appendix*, Fig. S6*B*). No differences in bactericidal activity were observed between STM-4/74 RibB^++^ and STM-4/74. While macrophages pretreated with IFN-γ acquired bactericidal activity against all *Salmonella* isolates (*SI Appendix*, Fig. S6*C*), MAIT cell antimicrobial activity in these in vitro cocultures was not directly correlated to the amount of IFN-γ found in supernatants (*SI Appendix*, Fig. S6*D*).

## Discussion

Here we investigated the ability of MAIT cells to recognize and respond to diverse invasive *S. enterica* serovars. We found that MAIT cells isolated from the blood of healthy individuals were not activated by exposure to invasive disease-associated *S.* Typhimurium sequence type 313 (ST313) lineage 2 strains. Our data demonstrated how *S.* Typhimurium ST313 lineage 2 evades MAIT cell recognition by overexpressing *ribB*, a bacterial gene encoding the RibB enzyme involved in the riboflavin pathway. Our results lead us to propose that this RibB-mediated mechanism provides an evolutionary advantage that allows invasive *S.* Typhimurium ST313 lineage 2 bacteria to escape cell immune responses by overexpressing a single riboflavin bacterial gene.

MR1-restricted MAIT cells are highly abundant in the gut mucosa (49), where they reach their final maturation upon recognition of vitamin B2 metabolites derived from gut commensals presented by MR1 expressing mucosal B cells (49). MAIT cells show antimicrobial activity in vivo and in vitro (50, 51) through MR1 dependent and independent interactions. Microorganisms must express the riboflavin biosynthesis pathway to be able to activate MAIT cells (29, 52). Mutations in key enzymes of the riboflavin biosynthetic pathway in both gram-positive and -negative bacteria can abrogate MAIT cell activation (33, 45). Different bacteria that possess the riboflavin biosynthetic pathway induce varying levels of MAIT stimulation (36, 37), possibly through the influences of the microenvironment on bacterial metabolism and antigen availability, or the known short half-life of the potent MAIT cell antigens, 5-OP-RU and 5-OE-RU (53). The ability of MAIT cells to recognize and respond to several isolates of the same pathogen may also vary to reflect bacterial metabolic differences. For example, riboflavin metabolism variation among clinical isolates of *Streptococcus pneumoniae* produces different measurable levels of riboflavin and FMN that correlate with differential activation of MAIT cells (38).

*Salmonella* spp. possess an active riboflavin biosynthetic pathway, which generates MAIT cell agonists (29, 33). MAIT cells recognize and kill *S.* Typhimurium-infected targets in vitro (32), and activated MAIT cells accumulate in murine lungs following intranasal infection with *S.* Typhimurium (26). However, bacterial lung clearance was independent of MAIT cells in this infection model, possibly due to the nonphysiological route of infection. While some mouse models of infection with *Salmonella* sequence type 313 strains have been published (14, 54), these models present limited utility as mice have very low frequencies and absolute numbers of MAIT cells compared with humans (28). Human studies demonstrated sustained MAIT cell activation and proliferation at the peak of infection with *S.* Typhi and *S.* Paratyphi (34, 35). Among immunocompetent humans, the clinical outcomes of infection by *S. enterica* spp. depend on the infecting serovar. Human-restricted typhoidal serovars, such as *S*. Typhi induce the most severe form of systemic disease, typhoid fever; while the broad-host *S.* Typhimurium sequence type 19 causes self-limiting gastroenteritis. The recently documented multidrug resistant *S.* Typhimurium ST313 clade causes the majority of iNTS cases among immunocompromised adults and malnourished young children living in sub-Saharan Africa.

Several bacterial factors have been reported to enhance invasiveness of *S*. Typhimurium ST313, suggesting a multifactorial adaptation of this African lineage to a systemic lifestyle. These include interference with the complement cascade (46), interference with dendritic cell (DC) function (43), reduced inflammasome activation (55), and dissemination through CD11b^+^ migratory DC (56), among others. In addition, Salmonella is able to evade the immune system through persistent asymtpomatic infections (57, 58).

Here we found that *S.* Typhi, *S*. Paratyphi A, and most *S.* Typhimurium pathovars potently elicited ex vivo MR1-dependent MAIT cell activation, but all tested isolates from the invasive *S.* Typhimurium ST313 lineage 2 barely induced cytokine secretion or CD69 up-regulation. This suboptimal activation was restricted to MAIT cells, as γδ cell activation was comparable across all isolates tested. We excluded differences in infection efficiency, MR1 expression, costimulatory cytokines (IL-12), the genetic sequence of the riboflavin-encoding enzymes of *S.* Typhimurium, and the presence of dominant inhibitory ligands as potential mechanisms for these results. Following comparative proteomic and transcriptomic analyses, we discovered that invasive *S.* Typhimurium ST313 lineage 2 pathovars escape MAIT cell recognition by overexpressing *ribB*, a bacterial gene encoding the riboflavin biosynthetic enzyme RibB. By overexpressing this single riboflavin gene in a sequence type 19 *S*. Typhimurium strain, we revealed that up-regulation of this single riboflavin gene was sufficient to abrogate MAIT cell responses.

Transcriptional control of the bacterial *ribB* gene is controlled by a conserved FMN riboswitch, which is located in the 5′ UTR of *ribB* (59) and is negatively regulated by FMN and other flavins at the transcriptional and translational levels in *E. coli* (60). The SroG small RNA (sRNA) is derived from the *ribB* 5′ leader sequence, although the function of this sRNA remains unknown (61). In addition to the riboswitch-mediated regulation, RibB expression is induced by growth in a low pH environment (62). In STM-D23580, both *sroG* and *ribB* are transcribed as a single transcript that is initiated from a single gene promoter which we identified previously (46).

While the precise molecular mechanism responsible for *ribB* overexpression remains to be established, a single noncoding nucleotide polymorphism in the promoter of the *pgtE* gene of the ST313 lineage 2 strain STM-D23580 is known to be responsible for high expression of the outer membrane PgtE virulence factor, which promotes bacterial survival and dissemination during mammalian infection (46). The lack of nucleotide differences between the promoter sequences of the *ribB* genes or the riboswitch of the two strains suggests that the high level of expression of *ribB* in STM-D23580 is caused by a novel and uncharacterized regulatory mechanism.

It has been proposed that genomic changes in ST313 isolates that confer altered metabolism and increased anaerobic metabolic capacity are linked to adaptation of the extraintestinal niche (14). Riboflavin and its derivatives are important cofactors for flavoproteins involved in cellular redox metabolism and several biochemical pathways, proposed to be essential for the metabolic adaptation of the ST313 clade. Riboflavin promotes intracellular microbial survival and virulence during in vivo infection with *Histoplasma capsulatum* and *Brucella abortus* (63, 64). In addition, accumulation of riboflavin is a candidate virulence factor in *Pseudogymnoascus destructans* skin infection (65).

The metabolomic measurements of the end products of the riboflavin pathway showed a correlation between lower levels of intracellular FMN and increased expression of RibB. Because FMN is a negative regulator of *ribB* gene expression (60), we speculate that the lower levels of intracellular FMN observed in the ST313 strains D23580 and D37712 are linked to the high levels of expression of RibB in these African *S.* Typhimurium strains.

Overall, our findings suggest that MAIT cells play a crucial role in defense against invasive *Salmonella* disease in humans and that evasion from MAIT cell recognition is a critical mechanism for the invasiveness of *S*. Typhimurium ST313 lineage 2 isolates. We propose that differences in MAIT cell activation may associate with distinct diseases caused by closely related microorganisms. The increased susceptibility of immunocompromised patients to the *S*. Typhimurium ST313 lineage 2 strains suggests that MAIT cells might play a particularly relevant role in the context of waning CD4^+^ T cell-mediated protective adaptive immunity, where protection relies mostly on the innate immune response. For example, following HIV coinfection and/or malnutrition, among individuals suffering from recurrent gut infections secondary to intestinal barrier dysfunction (66, 67), microbiota dysbiosis (68, 69), and multiple innate and adaptive immune defects (70). Our findings may be of major relevance during the initial phase of infection, in the gut, where it is expected that resident immune cells such as MAIT cells should prevent systemic infection by encountering and responding rapidly to bacterial signals. We propose that the ability of MAIT cells to target gastrointestinal pathogens represents a key immunological evolutionary bottleneck that has been effectively countered by *Salmonella*, resulting in the current epidemic of invasive disease in Africa.

## Methods

### Bacterial Strains and Preparation of Stocks.

This study included representative strains of *S. enterica* serovar Typhimurium, from both the sequence type 19 and the sequence type 313. *S.* Typhi and *S.* Paratyphi A serovars were utilized as comparative Typhoidal invasive strains, while *E. coli* (DH5α) was used as unrelated bacterial control. *SI Appendix*, Table S1 lists bacterial strains used in this study.

Overnight bacterial cultures from a single colony origin were used to inoculate Luria broth (LB) Lennox broth (Sigma) supplemented with sucrose (Sigma) at a final concentration of 10%. Inoculated cultures were incubated at 37 °C under constant shaking for ∼3 h, until reaching midlogarithmic phase. Bacterial aliquots were prepared and immediately frozen at −80 °C for long-term storage. Bacterial viability of frozen aliquots was monitored periodically in order to maintain experimental reproducibility. The number of viable colony-forming units was determined with the Miles and Misra method, by plating 10-fold dilutions of the bacterial suspension onto LB Lennox agar (Sigma). On the day of the experiment, a single aliquot was thawed, washed twice with phosphate-buffered saline (PBS), and resuspended in RPMI 1640 media to obtain the desired MOI.

In the case of bacterial supernatants, these were taken from late exponential phase cultures, grown from a single colony following 18-h incubation under constant shaking. Supernatants were filter sterilized before using.

### Construction of *S*. Typhimurium 4/74 pP_L_-*ribB*.

To construct pP_L_-*ribB*, the *ribB* gene was amplified from genomic DNA of *S.* Typhimurium 4/74 using primers *ribB*_FW and *ribB*_RV. The PCR product was used for a linear amplification reaction with plasmid pJV300 (pP_L_) using Phusion DNA polymerase (New England Biolabs), and the resulting product was digested with *Dpn*I. The plasmid was transformed into *E. coli* TOP10 and selected on LB plates supplemented with 100 μg/mL ampicillin. Plasmid presence was confirmed by PCR and DNA sequencing using oligonucleotides pPL_Seq_FW and pPL_Seq_RV. The pP_L_-*ribB* plasmid was subsequently purified and transformed into *S.* Typhimurium 4/74. *SI Appendix*, Table S2 lists plasmids and oligonucleotides used in this study.

### Isolation of Human Cells from Peripheral Blood from Healthy Volunteers in the United Kingdom.

Leukocyte reduction system cones were obtained from the UK National Blood Centre with informed consent following local ethical guidelines (National Health Service Blood and Transplant [NHSTB] account T293). Blood was diluted in PBS and separated by gradient centrifugation using Lymphoprep (AxisShield). PBMCs were collected from the interface, washed with PBS, resuspended in complete medium, and counted. Complete medium used throughout was RPMI 1640 (Sigma), supplemented with 10% heat-inactivated fetal calf serum (FCS, Sigma), 2 mM l-glutamine, 1% nonessential amino acids, and 1% sodium pyruvate (all from Gibco).

### Isolation of PBMCs from Healthy and HIV-Infected Volunteers in Malawi.

Blood samples were obtained at Queen Elizabeth Central Hospital (Blantyre, Malawi) following local ethical guidelines. Samples were deidentified prior to use. Our study was approved by the Malawi College of Medicine Research Ethics Committee, Malawi COMREC, P09/17/2284). Adults presenting for HIV testing at the voluntary testing clinic, the HIV outpatient clinic, and the medical inpatient wards at the Queen Elizabeth Central Hospital were recruited. Based on the use of antiretroviral therapy, these patients were classified as ART naïve (without) or ART treated (with). Upon appropriate consent and medical authorization, a blood sample was collected and PBMCs were isolated and used in ex vivo infection assays, as described below.

### Ex Vivo Infection Assays with PBMCs.

PBMCs were seeded in 96-well round-bottom plates (5 to 8 × 10^5^ cells per well) and infected with the different *Salmonella* strains from frozen midlog phase stocks, at the indicated MOI. Upon 80-min incubation at 37 °C, 100 μg/mL gentamicin (Lonza) was added to kill extracellular bacteria. A total of 200 μg/mL of gentamicin was required for the experiments carried out in Malawi using ST313 strains from lineages 1 and 2.

At 180 min postinfection, 5 ng/mL brefeldin A (BioLegend) solution was added to every well in order to achieve accumulation of intracellular cytokines. Samples were incubated overnight at 37 °C for no more than 15 h.

For MR1 blocking experiments, infection was performed in the presence of 30 μg/mL MR1 blocking antibody 26.5 (42) or mouse IgG2a isotype control (ATCC). The MAIT agonists 5-A-RU was synthesized as described in ref. 71 and 1 μg/mL was combined to 50 μM MG (Sigma).

### Assessment of Cytokine Production and MAIT Cell Activation by Flow Cytometry.

Following incubation in the presence of brefeldin A, cells were harvested, washed, and stained with a viability dye (live/dead Zombie Aqua, BioLegend) for 20 min. Fixation was performed for 30 min at 4 °C using the Foxp3 Fixation/Permeabilization buffers (eBioscience). Fixed cells were permeabilized and stained with an antibody mixture for 40 min at room temperature, washed, and stored protected from light at 4 °C in PBS with 0.5% bovine serum albumin (BSA) (FACS buffer) until acquisition. The following antibodies were used for extracellular and intracellular staining as two different panels: anti-CD3 Alexa700 (clone UCHT1; BioLegend), anti-CD3 PerCp Cy5.5 (clone UCHT1; BioLegend), anti-CD4 APCef780 (clone RPA-T4; eBioscience), anti-CD4 Alexa700 (clone RPA-T4; BioLegend), anti-CD8 BV785 (clone RPA-T8; BioLegend), anti-TCR γ/δ APC (clone B1; BioLegend), anti-CD161 BV605 (clone HP-3G10; BioLegend), anti-CD161 BV421 (clone HP-3G10; BioLegend), anti-Vα7.2 PE (clone 3C10; BioLegend), anti-Vα7.2 PE-Cy7 (clone 3C10; BioLegend), anti-CD69 FITC (clone FN50; BioLegend), anti TNF- α PECy7 (clone Mab11; BioLegend), anti TNF-α APC (clone Mab11; BioLegend), anti-IFN-γ FITC (clone 4S.B3; BioLegend), and anti-IFN-γ PE Dazzle (clone 4S.B3; BioLegend). Samples from the United Kingdom were acquired on a FortessaX20 (BD), while samples from the case-control study in Malawi were acquired on a LSR Fortessa cytometer (BD). All data were analyzed with the same gating strategy on FlowJo (v.10.6.1).

### Unsupervised Analysis of Flow Cytometry Data.

Two dimensionality reduction methods based on a neighboring graph approach were implemented, *t*-SNE (72) and UMAP (73). *t*-SNE algorithm was performed on the Cytofkit platform (74) using up to 5,000 cells from each sample. UMAP was run as a plugin on FlowJo (v.10.4.1) using 15 nearest neighbors, a minimum distance of 0.5, and Euclidean distance for selected parameters. Files with .fcs extension from related experimental conditions were concatenated before UMAP analysis.

### Coculture of *Salmonella*-Infected Monocyte-Derived Dendritic Cells and Purified T Cells.

MoDCs were obtained from PBMCs by enrichment of CD14^+^ monocytes using magnetic beads (Miltenyi). Differentiation was achieved with recombinant human GM-CSF (40 ng/mL) and human IL-4 (40 ng/mL), both from PeproTech. After 5 d, MoDCs were infected with violet-labeled (CellTracker, Life Technologies) *Salmonella* strains, either STM-D23580 or STM-LT2, at an MOI of 10, as reported elsewhere (43).

At 6 h postinfection, *Salmonella*-containing MoDCs were FACS sorted as single cells. Sorted MoDCs were cocultured with magnetically enriched (Miltenyi) CD3^+^ T cells obtained from the same donor, at a ratio of one MoDC per six T cells. Following 12-h incubation in the presence of brefeldin A, T cells were harvested and stained for intracellular cytokines as described above.

### Coculture of *Salmonella*-Infected MoDCs and Expanded MAIT Cells.

Human MoDCs were obtained as described above. Human MAIT cells were isolated by sorting CD2 MACS-enriched (Miltenyi) leukocytes with CD161 and Vα7.2 antibodies (BioLegend). MAIT cells were grown for around 6 wk in complete RPMI media supplemented with IL-2, as described elsewhere in ref. 71.

A total of 40,000 MoDCs and 20,000 MAIT cells (2:1 ratio) were seeded in 96-well flat-bottom plates and infected at MOI of 3.5. After 80 min, 100 μg/mL gentamicin was added and supernatants were harvested following 26-h incubation. IL-12 p70 was measured by ELISA (R&D Systems) in triplicates and following manufacturer’s instructions.

### Coculture of *Salmonella*-Infected Monocyte-Derived Macrophages and Expanded MAIT Cells.

Monocytes were obtained from leukocyte reduction system cones by enrichment of CD14^+^ cells using magnetic beads (Miltenyi), according to manufacturer’s protocol. Monocytes were seeded in 24-well plates (450,000 to 500,000 cells/well) and differentiated into macrophages using recombinant human M-CSF at 100 ng/mL (PeproTech). After 6 d of incubation at 37 °C and 5% CO_2_, the adherent macrophages were carefully washed to remove M-CSF-containing media and fresh antibiotic-free medium was added. Next, macrophages were infected with the different *Salmonella* strains at a MOI of 15. After 30 min postinfection, cells were washed and incubated for an additional 30 min with 100 μg/mL of gentamicin-containing medium to kill any remaining extracellular bacteria. At 1 h postinfection, macrophages were washed again and MAIT cells were added (ratio of one MAIT cell per five macrophages) into the respective wells. From this point onwards, media contained gentamicin at 30 μg/mL as a maintenance dose. Cells were incubated until completing 6 h postinfection before being washed twice with PBS and lysed with 2% saponin. The number of intracellular viable bacterial cfus was determined with the Miles and Misra method as described above. IFN-γ in the supernatants was measured with a commercial ELISA (BD-Pharmingen), as per manufacturer instructions.

### MR1 Overexpressing Cell Line.

THP-1 cells were transduced with an MR1-encoding lentivirus (34). MR1-overexpressing cells were seeded in 96-well flat-bottom plates and incubated overnight in the presence of 50 μL of supernatants from bacterial cultures at late exponential phase, or in the presence of 5-A-RU as positive control. Cells were harvested, washed, and stained for surface expression of MR1 (clone 26.5; BioLegend) by flow cytometry. Expression of MHC-I (clone G46-2.6, BD Biosciences) was also monitored as unrelated control.

### Transcriptomic and Proteomic Analyses of Riboflavin Enzymes.

RNA sequencing (RNA-seq) and proteomic data for genes involved in the riboflavin biosynthetic pathway were extracted from recent work (44). Briefly, a differential expression comparative analysis between strains STM-D23580 and STM-4/74 was performed at the transcriptomic level in five in vitro infection-relevant conditions: ESP, anaerobic growth, NonSPI2 (SPI2-noninducing phosphate carbon nitrogen minimal medium [PCN]), InSPI2 (SPI2-inducing PCN), and inside murine RAW264.7 macrophages (ATCC, TIB-71). Specific details about growing bacteria in these conditions had been previously described (40, 75). For a comparative proteomic analysis, bacteria were grown to ESP in the LB-rich medium.

The RNA-seq-based comparative approach between STM-D23580 and STM-4/74 was based on Voom/Limma analysis from three different biological replicates for each strain. A detailed pipeline for the analysis can be found in Canals et al. (44).

Proteomic data for strains STM-D23580 and STM-4/74 were obtained using a liquid chromatography with tandem mass spectrometry (LC-MS/MS, Q Exactive Orbitrap, 4-h reversed phase C18 gradient) platform. Samples included six biological replicates for each strain. Label-free quantification and differential expression analyses between the two strains were performed using Progenesis QI software (Nonlinear Dynamics) (44).

### Measurement of Riboflavin, FMN, and FAD in Supernatants and Pellets of Bacterial Cultures.

Bacterial pellets and supernatants from early stationary phase cultures (OD_600_ = ∼2) were prepared in triplicate and frozen at −80 °C. For extraction of cellular flavins, pellets were resuspended in 100 μL of 100 mM ammonium formate, 100 mM formic acid, 25% (vol/vol) methanol, and heated at 80 °C for 10 min. Insoluble material was removed by centrifugation. For analysis, 5 μL of this material or the culture supernatants was separated by HPLC on a Dionex UPLC system with a Kinetex C18 column (Phenomenex; 1.7 μm, 150 × 2.1 mm). Separation was achieved at 45 °C and 0.2 mL/min isocratically using 20 mM potassium phosphate buffer (pH 2.5) with 25% methanol (vol/vol) over 8 min followed by a 1-min wash step in 100% methanol. Flavins were detected with fluorescence (450-nm excitation, 520-nm emission) and peaks were quantified by comparison to known standards. For normalization the total picomole of flavin for each culture was divided by the measured OD_600_ of the cultures to give a final pmol/OD_600_ value.

### Statistical Analysis.

Statistical analyses were performed using GraphPad Prism8 (GraphPad Software). Differences among groups were determined by paired one-way, two-way ANOVA, or Kruskal–Wallis as appropriate. Post hoc corrections were applied, Dunnett’s, or Dunn’s for comparisons to a control dataset, and Bonferroni’s, Tukey’s, or Sidak’s for comparisons of selected pair tests, as appropriate. A *P* value <0.05 was considered statistically significant (*P < 0.05, **P < 0.01, ***P < 0.001, and ****P < 0.0001).

## Supplementary Material

Supplementary File

## Data Availability

All data have been made available in the manuscript.

## References

[1] Feasey N. A., Dougan G., Kingsley R. A., Heyderman R. S., Gordon M. A., Invasive non-typhoidal salmonella disease: An emerging and neglected tropical disease in Africa. Lancet 379, 2489–2499 (2012). 22587967 10.1016/S0140-6736(11)61752-2 PMC3402672

[2] Keestra-Gounder A. M., Tsolis R. M., Bäumler A. J., Now you see me, now you don’t: The interaction of Salmonella with innate immune receptors. Nat. Rev. Microbiol. 13, 206–216 (2015). 25749454 10.1038/nrmicro3428

[3] Achtman M..; S. Enterica MLST Study Group, Multilocus sequence typing as a replacement for serotyping in Salmonella enterica. PLoS Pathog. 8, e1002776 (2012). 22737074 10.1371/journal.ppat.1002776 PMC3380943

[4] Kingsley R. A.., Epidemic multiple drug resistant Salmonella Typhimurium causing invasive disease in sub-Saharan Africa have a distinct genotype. Genome Res. 19, 2279–2287 (2009). 19901036 10.1101/gr.091017.109 PMC2792184

[5] Reddy E. A., Shaw A. V., Crump J. A., Community-acquired bloodstream infections in Africa: A systematic review and meta-analysis. Lancet Infect. Dis. 10, 417–432 (2010). 20510282 10.1016/S1473-3099(10)70072-4 PMC3168734

[6] Okoro C. K.., Intracontinental spread of human invasive Salmonella Typhimurium pathovariants in sub-Saharan Africa. Nat. Genet. 44, 1215–1221 (2012). 23023330 10.1038/ng.2423 PMC3491877

[7] Stanaway J. D..; GBD 2017 Non-Typhoidal Salmonella Invasive Disease Collaborators, The global burden of non-typhoidal salmonella invasive disease: A systematic analysis for the global burden of disease study 2017. Lancet Infect. Dis. 19, 1312–1324 (2019). 31562022 10.1016/S1473-3099(19)30418-9 PMC6892270

[8] Berkley J. A.., HIV infection, malnutrition, and invasive bacterial infection among children with severe malaria. Clin. Infect. Dis. 49, 336–343 (2009). 19548833 10.1086/600299 PMC2853703

[9] Biggs H. M.., Invasive Salmonella infections in areas of high and low malaria transmission intensity in Tanzania. Clin. Infect. Dis. 58, 638–647 (2014). 24336909 10.1093/cid/cit798 PMC3922215

[10] Gordon M. A., Salmonella infections in immunocompromised adults. J. Infect. 56, 413–422 (2008). 18474400 10.1016/j.jinf.2008.03.012

[11] Gordon M. A.., Non-typhoidal salmonella bacteraemia among HIV-infected Malawian adults: High mortality and frequent recrudescence. AIDS 16, 1633–1641 (2002). 12172085 10.1097/00002030-200208160-00009

[12] Ashton P. M.., Public health surveillance in the UK revolutionises our understanding of the invasive Salmonella Typhimurium epidemic in Africa. Genome Med. 9, 92 (2017). 29084588 10.1186/s13073-017-0480-7 PMC5663059

[13] Almeida F.., Multilocus sequence typing of Salmonella Typhimurium reveals the presence of the highly invasive ST313 in Brazil. Infect. Genet. Evol. 51, 41–44 (2017). 28288927 10.1016/j.meegid.2017.03.009

[14] Okoro C. K.., Signatures of adaptation in human invasive Salmonella Typhimurium ST313 populations from sub-Saharan Africa. PLoS Negl. Trop. Dis. 9, e0003611 (2015). 25803844 10.1371/journal.pntd.0003611 PMC4372345

[15] Parsons B. N.., Invasive non-typhoidal Salmonella typhimurium ST313 are not host-restricted and have an invasive phenotype in experimentally infected chickens. PLoS Negl. Trop. Dis. 7, e2487 (2013). 24130915 10.1371/journal.pntd.0002487 PMC3794976

[16] Griffin A. J., McSorley S. J., Development of protective immunity to Salmonella, a mucosal pathogen with a systemic agenda. Mucosal Immunol. 4, 371–382 (2011). 21307847 10.1038/mi.2011.2 PMC4084725

[17] Hess J., Ladel C., Miko D., Kaufmann S. H., Salmonella typhimurium aroA- infection in gene-targeted immunodeficient mice: Major role of CD4+ TCR-alpha beta cells and IFN-gamma in bacterial clearance independent of intracellular location. J. Immunol. 156, 3321–3326 (1996). 8617956

[18] Lee S.-J., Dunmire S., McSorley S. J., MHC class-I-restricted CD8 T cells play a protective role during primary Salmonella infection. Immunol. Lett. 148, 138–143 (2012). 23089550 10.1016/j.imlet.2012.10.009 PMC3540194

[19] Napolitani G.., Clonal analysis of Salmonella-specific effector T cells reveals serovar-specific and cross-reactive T cell responses. Nat. Immunol. 19, 742–754 (2018). 29925993 10.1038/s41590-018-0133-z

[20] Reynolds C. J.., The serodominant secreted effector protein of Salmonella, SseB, is a strong CD4 antigen containing an immunodominant epitope presented by diverse HLA class II alleles. Immunology 143, 438–446 (2014). 24891088 10.1111/imm.12327 PMC4212957

[21] Wahid R., Fresnay S., Levine M. M., Sztein M. B., Cross-reactive multifunctional CD4+ T cell responses against Salmonella enterica serovars Typhi, Paratyphi A and Paratyphi B in humans following immunization with live oral typhoid vaccine Ty21a. Clin. Immunol. 173, 87–95 (2016). 27634430 10.1016/j.clim.2016.09.006 PMC5322816

[22] Sheikh A.., Interferon-γ and proliferation responses to Salmonella enterica serotype Typhi proteins in patients with S. Typhi Bacteremia in Dhaka, Bangladesh. PLoS Negl. Trop. Dis. 5, e1193 (2011). 21666798 10.1371/journal.pntd.0001193 PMC3110156

[23] Pennington S. H.., Oral typhoid vaccination with live-attenuated Salmonella Typhi strain Ty21a generates ty21a-responsive and heterologous influenza virus-responsive CD4+ and CD8+ T cells at the human intestinal mucosa. J. Infect. Dis. 213, 1809–1819 (2016). 26810369 10.1093/infdis/jiw030 PMC4857474

[24] Nyirenda T. S.., Sequential acquisition of T cells and antibodies to nontyphoidal Salmonella in Malawian children. J. Infect. Dis. 210, 56–64 (2014). 24443544 10.1093/infdis/jiu045 PMC4054899

[25] Brigl M., Bry L., Kent S. C., Gumperz J. E., Brenner M. B., Mechanism of CD1d-restricted natural killer T cell activation during microbial infection. Nat. Immunol. 4, 1230–1237 (2003). 14578883 10.1038/ni1002

[26] Chen Z.., Mucosal-associated invariant T-cell activation and accumulation after in vivo infection depends on microbial riboflavin synthesis and co-stimulatory signals. Mucosal Immunol. 10, 58–68 (2017). 27143301 10.1038/mi.2016.39

[27] Davies A.., Infection-induced expansion of a MHC Class Ib-dependent intestinal intraepithelial gammadelta T cell subset. J. Immunol. 172, 6828–6837 (2004). 15153501 10.4049/jimmunol.172.11.6828

[28] Lantz O., Legoux F., MAIT cells: An historical and evolutionary perspective. Immunol. Cell Biol. 96, 564–572 (2018). 29363173 10.1111/imcb.1034

[29] Kjer-Nielsen L.., MR1 presents microbial vitamin B metabolites to MAIT cells. Nature 491, 717–723 (2012). 23051753 10.1038/nature11605

[30] Godfrey D. I., Koay H.-F., McCluskey J., Gherardin N. A., The biology and functional importance of MAIT cells. Nat. Immunol. 20, 1110–1128 (2019). 31406380 10.1038/s41590-019-0444-8

[31] Ussher J. E.., CD161++ CD8+ T cells, including the MAIT cell subset, are specifically activated by IL-12+IL-18 in a TCR-independent manner. Eur. J. Immunol. 44, 195–203 (2014). 24019201 10.1002/eji.201343509 PMC3947164

[32] Reantragoon R.., Structural insight into MR1-mediated recognition of the mucosal associated invariant T cell receptor. J. Exp. Med. 209, 761–774 (2012). 22412157 10.1084/jem.20112095 PMC3328369

[33] Corbett A. J.., T-cell activation by transitory neo-antigens derived from distinct microbial pathways. Nature 509, 361–365 (2014). 24695216 10.1038/nature13160

[34] Howson L. J.., MAIT cell clonal expansion and TCR repertoire shaping in human volunteers challenged with Salmonella Paratyphi A. Nat. Commun. 9, 253 (2018). 29343684 10.1038/s41467-017-02540-x PMC5772558

[35] Salerno-Goncalves R.., Challenge of humans with wild-type *Salmonella enterica* serovar Typhi elicits changes in the activation and homing characteristics of mucosal-associated invariant T cells. Front. Immunol. 8, 398 (2017). 28428786 10.3389/fimmu.2017.00398 PMC5382150

[36] Schmaler M.., Modulation of bacterial metabolism by the microenvironment controls MAIT cell stimulation. Mucosal Immunol. 11, 1060–1070 (2018). 29743612 10.1038/s41385-018-0020-9

[37] Tastan C.., Tuning of human MAIT cell activation by commensal bacteria species and MR1-dependent T-cell presentation. Mucosal Immunol. 11, 1591–1605 (2018). 30115998 10.1038/s41385-018-0072-x PMC6279574

[38] Hartmann N.., Riboflavin metabolism variation among clinical isolates of Streptococcus pneumoniae results in differential activation of mucosal-associated invariant T cells. Am. J. Respir. Cell Mol. Biol. 58, 767–776 (2018). 29356555 10.1165/rcmb.2017-0290OC PMC6002660

[39] Becht E.., Dimensionality reduction for visualizing single-cell data using UMAP. Nat. Biotechnol. 37, 38–44 (2018). 10.1038/nbt.4314 30531897

[40] Kröger C.., An infection-relevant transcriptomic compendium for Salmonella enterica serovar Typhimurium. Cell Host Microbe 14, 683–695 (2013). 24331466 10.1016/j.chom.2013.11.010

[41] Juno J. A., Phetsouphanh C., Klenerman P., Kent S. J., Perturbation of mucosal-associated invariant T cells and iNKT cells in HIV infection. Curr. Opin. HIV AIDS 14, 77–84 (2019). 30585802 10.1097/COH.0000000000000526

[42] Huang S.., Evidence for MR1 antigen presentation to mucosal-associated invariant T cells. J. Biol. Chem. 280, 21183–21193 (2005). 15802267 10.1074/jbc.M501087200

[43] Aulicino A.., Invasive Salmonella exploits divergent immune evasion strategies in infected and bystander dendritic cell subsets. Nat. Commun. 9, 4883 (2018). 30451854 10.1038/s41467-018-07329-0 PMC6242960

[44] Canals R.., Adding function to the genome of African Salmonella Typhimurium ST313 strain D23580. PLoS Biol. 17, e3000059 (2019). 30645593 10.1371/journal.pbio.3000059 PMC6333337

[45] Soudais C.., In vitro and in vivo analysis of the gram-negative bacteria-derived riboflavin precursor derivatives activating mouse MAIT cells. J. Immunol. 194, 4641–4649 (2015). 25870247 10.4049/jimmunol.1403224

[46] Hammarlöf D. L.., Role of a single noncoding nucleotide in the evolution of an epidemic African clade of *Salmonella*. Proc. Natl. Acad. Sci. U.S.A. 115, E2614–E2623 (2018). 29487214 10.1073/pnas.1714718115 PMC5856525

[47] Battley E. H., Escherichia coli and Salmonella Typhimurium. Cellular and molecular biology, volume 1; volume 2. Frederick C. Neidhardt, John L. Ingraham, Boris Magasanik, K. Brooks Low, Moselio Schaechter, H. Edwin Umbarger. Q. Rev. Biol. 63, 463–464 (1988).

[48] Rollenhagen C., Bumann D., Salmonella enterica highly expressed genes are disease specific. Infect. Immun. 74, 1649–1660 (2006). 16495536 10.1128/IAI.74.3.1649-1660.2006 PMC1418657

[49] Treiner E.., Selection of evolutionarily conserved mucosal-associated invariant T cells by MR1. Nature 422, 164–169 (2003). 12634786 10.1038/nature01433

[50] Meermeier E. W., Harriff M. J., Karamooz E., Lewinsohn D. M., MAIT cells and microbial immunity. Immunol. Cell Biol. 96, 607–617 (2018). 29451704 10.1111/imcb.12022 PMC6045460

[51] Salou M., Franciszkiewicz K., Lantz O., MAIT cells in infectious diseases. Curr. Opin. Immunol. 48, 7–14 (2017). 28750261 10.1016/j.coi.2017.07.009

[52] Le Bourhis L.., Antimicrobial activity of mucosal-associated invariant T cells. Nat. Immunol. 11, 701–708 (2010). 20581831 10.1038/ni.1890

[53] Mak J. Y.., Stabilizing short-lived Schiff base derivatives of 5-aminouracils that activate mucosal-associated invariant T cells. Nat. Commun. 8, 14599 (2017). 28272391 10.1038/ncomms14599 PMC5344979

[54] Yang J.., Characterization of the invasive, multidrug resistant non-typhoidal Salmonella strain D23580 in a murine model of infection. PLoS Negl. Trop. Dis. 9, e0003839 (2015). 26091096 10.1371/journal.pntd.0003839 PMC4474555

[55] Carden S., Okoro C., Dougan G., Monack D., Non-typhoidal Salmonella Typhimurium ST313 isolates that cause bacteremia in humans stimulate less inflammasome activation than ST19 isolates associated with gastroenteritis. Pathog. Dis. 73, ftu023 (2015). 25808600 10.1093/femspd/ftu023 PMC4399442

[56] Carden S. E.., Pseudogenization of the secreted effector gene sseI confers rapid systemic dissemination of S. Typhimurium ST313 within migratory dendritic cells. Cell Host Microbe 21, 182–194 (2017). 28182950 10.1016/j.chom.2017.01.009 PMC5325708

[57] Kurtz J. R., Goggins J. A., McLachlan J. B., Salmonella infection: Interplay between the bacteria and host immune system. Immunol. Lett. 190, 42–50, 10.1016/j.imlet.2017.07.006 (2017). 28720334 PMC5918639

[58] Kurtz J. R., Salmonella Persistence and Host Immunity Are Dictated by the Anatomical Microenvironment. Inf. and Immunity, 10.1128/IAI.00026-20 (2020). PMC7375767 32393507

[59] Gelfand M. S., Mironov A. A., Jomantas J., Kozlov Y. I., Perumov D. A., A conserved RNA structure element involved in the regulation of bacterial riboflavin synthesis genes. Trends Genet. 15, 439–442 (1999). 10529804 10.1016/s0168-9525(99)01856-9

[60] Pedrolli D.., The ribB FMN riboswitch from Escherichia coli operates at the transcriptional and translational level and regulates riboflavin biosynthesis. FEBS J. 282, 3230–3242 (2015). 25661987 10.1111/febs.13226

[61] Vogel J.., RNomics in Escherichia coli detects new sRNA species and indicates parallel transcriptional output in bacteria. Nucleic Acids Res. 31, 6435–6443 (2003). 14602901 10.1093/nar/gkg867 PMC275561

[62] Stancik L. M.., pH-dependent expression of periplasmic proteins and amino acid catabolism in Escherichia coli. J. Bacteriol. 184, 4246–4258 (2002). 12107143 10.1128/JB.184.15.4246-4258.2002 PMC135203

[63] Bonomi H. R.., An atypical riboflavin pathway is essential for Brucella abortus virulence. PLoS One 5, e9435 (2010). 20195542 10.1371/journal.pone.0009435 PMC2828483

[64] Garfoot A. L., Zemska O., Rappleye C. A., Histoplasma capsulatum depends on de novo vitamin biosynthesis for intraphagosomal proliferation. Infect. Immun. 82, 393–404 (2014). 24191299 10.1128/IAI.00824-13 PMC3911860

[65] Flieger M.., Vitamin B2 as a virulence factor in Pseudogymnoascus destructans skin infection. Sci. Rep. 6, 33200 (2016). 27620349 10.1038/srep33200 PMC5020413

[66] Roche J. K., Cabel A., Sevilleja J., Nataro J., Guerrant R. L., Enteroaggregative Escherichia coli (EAEC) impairs growth while malnutrition worsens EAEC infection: A novel murine model of the infection malnutrition cycle. J. Infect. Dis. 202, 506–514 (2010). 20594107 10.1086/654894 PMC2919845

[67] Yu J.., Environmental enteric dysfunction includes a broad spectrum of Inflammatory responses and epithelial repair processes. Cell. Mol. Gastroenterol. Hepatol. 2, 158–174.e1 (2015). 26973864 10.1016/j.jcmgh.2015.12.002 PMC4769221

[68] Subramanian S.., Persistent gut microbiota immaturity in malnourished Bangladeshi children. Nature 510, 417–421 (2014). 24896187 10.1038/nature13421 PMC4189846

[69] Smith M. I.., Gut microbiomes of Malawian twin pairs discordant for kwashiorkor. Science 339, 548–554 (2013). 23363771 10.1126/science.1229000 PMC3667500

[70] Bourke C. D., Berkley J. A., Prendergast A. J., Immune dysfunction as a cause and consequence of malnutrition. Trends Immunol. 37, 386–398 (2016). 27237815 10.1016/j.it.2016.04.003 PMC4889773

[71] Salio M.., Activation of human mucosal-associated invariant T cells induces CD40L-dependent maturation of monocyte-derived and primary dendritic cells. J. Immunol. 199, 2631–2638 (2017). 28877992 10.4049/jimmunol.1700615 PMC5632842

[72] van der Maaten L. J. P., Hinton G. E., Visualizing high-dimensional data using t-SNE. J. Mach. Learn. Res. 9, 2579–2605 (2008).

[73] McInnes L., Healy J., Saul N., Großberger L., UMAP: Uniform manifold approximation and projection. J. Open Source Softw. 3, 861 (2018).

[74] Chen H.., Cytofkit: A bioconductor package for an integrated mass cytometry data analysis pipeline. PLOS Comput. Biol. 12, e1005112 (2016). 27662185 10.1371/journal.pcbi.1005112 PMC5035035

[75] Srikumar S.., RNA-seq brings new insights to the intra-macrophage transcriptome of Salmonella Typhimurium. PLoS Pathog. 11, e1005262 (2015). 26561851 10.1371/journal.ppat.1005262 PMC4643027

