# Supplementary material for: Evasion of MAIT cell recognition by the African Salmonella Typhimurium ST313 pathovar that causes invasive disease
Source: Proc Natl Acad Sci U S A. 2020 Aug 25;117(34):20717–28. doi: 10.1073/pnas.2007472117 (PMC7456131; doi:10.1073/pnas.2007472117)
Supplement: Supplementary File [file pnas.2007472117.sapp.pdf]

Supplementary Information for:

Evasion of MAIT cell recognition by the African Salmonella Typhimurium ST313 pathovar that causes invasive disease

Preciado-Llanes *et al*.

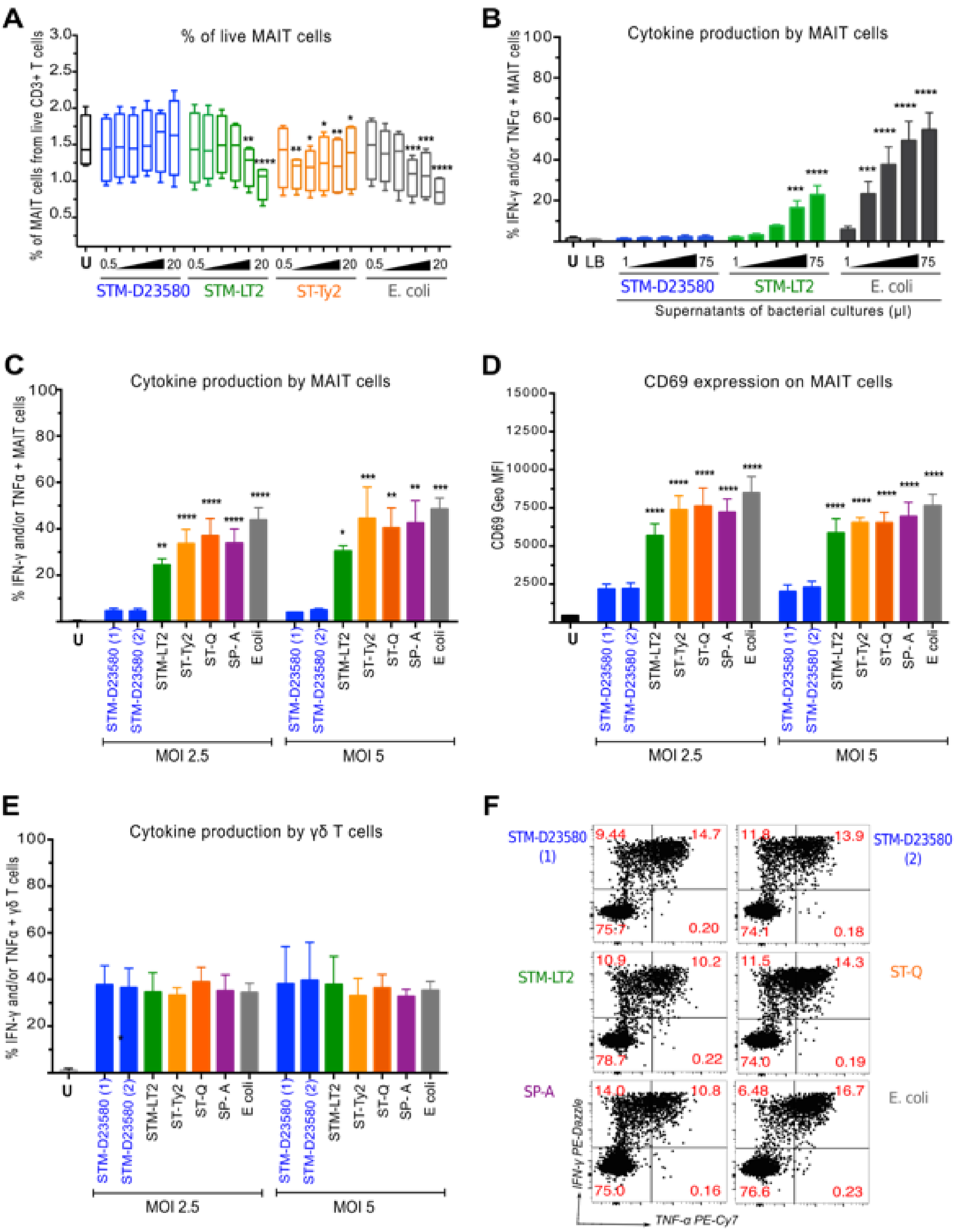

Figure S1. STM-D23580 escapes from MAIT cell recognition without affecting cell viability and without reducing γδ T cell activation. (A) PBMC were left unstimulated (U) or were infected with a variety of *Salmonella* strains at increasing MOI, from 0.5 to 20 bacteria per cell. *E coli* was included as positive control. Frequencies of MAIT cells from gated live CD3+ T cells are plotted. Data represented as box-and-whisker plot, two-way

ANOVA + Dunnet's, *n*=4. **(B)** Percentage of TNF-α and/or IFN-γ producing MAIT cells when stimulated with increasing amounts of bacterial culture supernatants, from 1 to 75 µL (final volume in well 250 µL). Data represented as mean ± SEM, two-way ANOVA + Dunnet's, *n*=4. **(C)** Percentage of TNF-α and/or IFN-γ producing MAIT cells, treated with bacterial strains at MOI of 2.5 and 5. Two STM-D23580 stocks were tested and are shown as (1) and (2). Data represented as mean ± SEM, one-way ANOVA + Dunnet's, *n*=5 for MOI 2.5 and *n*=3 for MOI 5. **(D)** Levels of CD69 expression on MAIT cells treated as in (C). Data represented as geometric mean ± SEM, one-way ANOVA + Dunnet's, *n*=5 for MOI 2.5 and *n*=3 for MOI 5. **(E)** Percentage of TNF-α and/or IFN-γ producing γδ T cells, treated with bacterial strains at MOI of 2.5 and 5. Data represented as mean ± SEM, one-way ANOVA + Dunnet's, *n*=5 for MOI 2.5 and *n*=3 for MOI 5. **(F)** Representative example of cytokine production by stimulated γδ T cells treated as in (E).

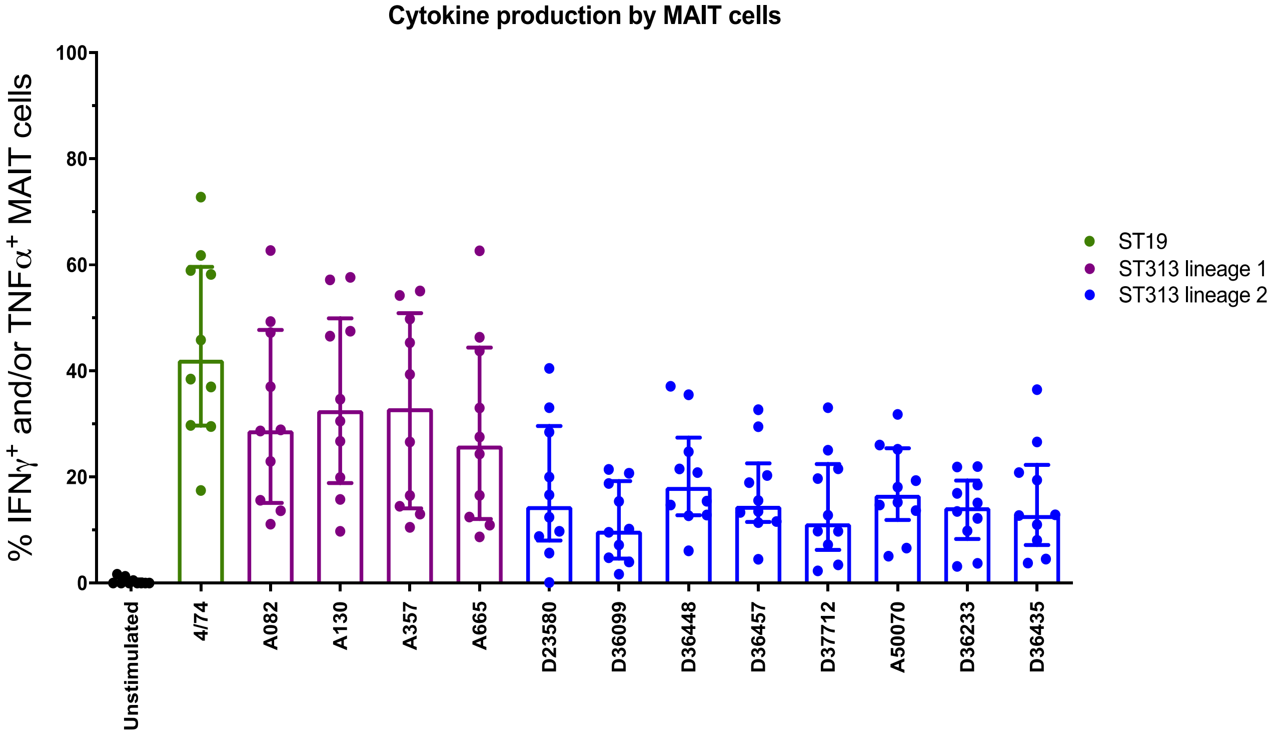

Figure S2. Characterisation of MAIT cell responses to *Salmonella spp*. in a cohort of healthy individuals living in Malawi.

PBMC were infected at MOI of 7 with various strains from sequence type 313 (ST313) lineages 1 and 2, or with the sequence type 19 (ST19) reference strain STM-4/74. Data represented as percentage of TNF-α and/or IFN-γ producing MAIT cells measured by intracellular flow cytometry staining. Median + IQR, *n*=10 for each group.

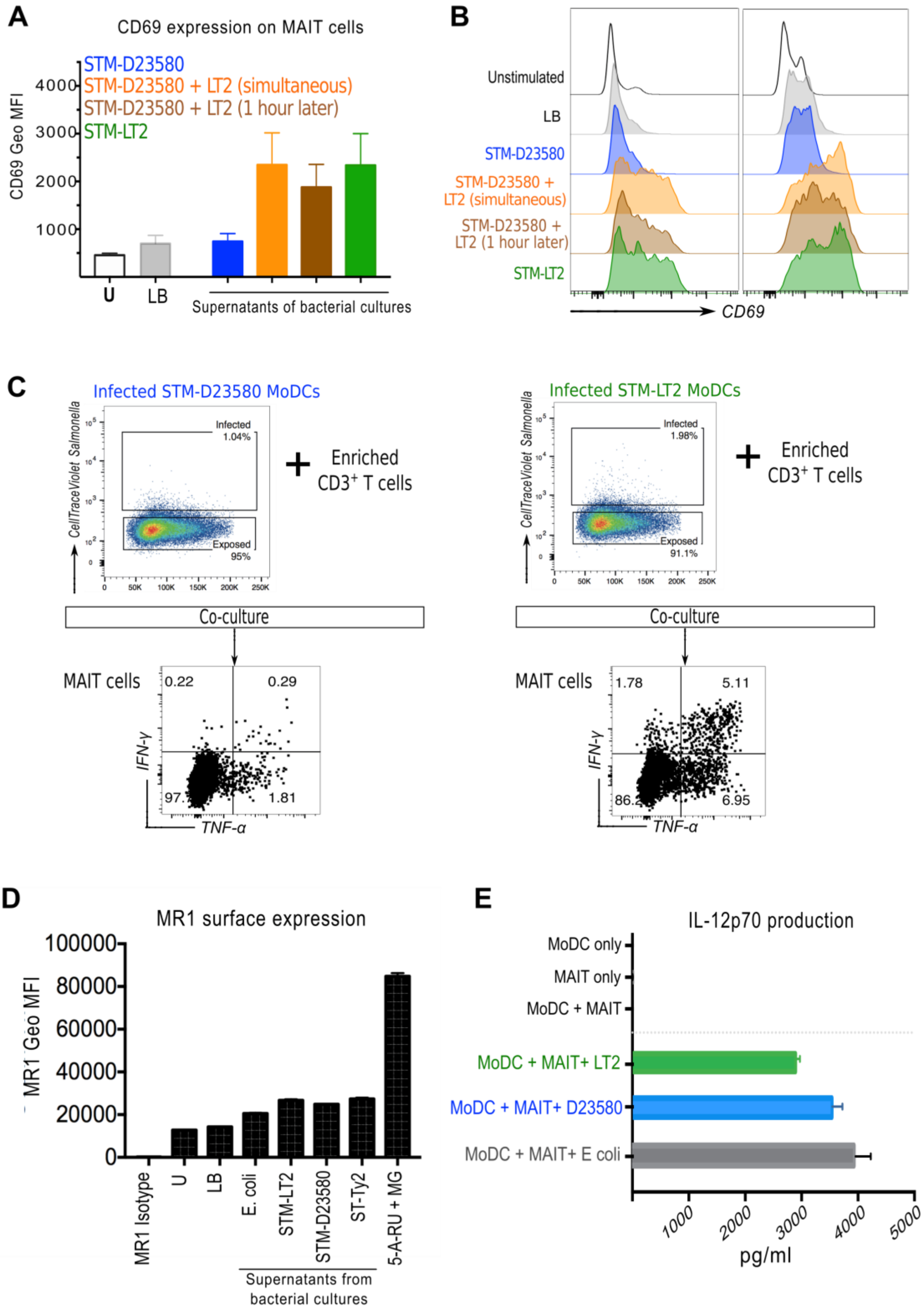

Figure S3. STM-D23580 does not interfere with MR1-dependent presentation or with IL-12p70p production. (A) CD69 expression on MAIT cells upon PBMC stimulation for 6 hours with 60 μL of STM-D23580 culture supernatant alone (blue bar) or in combination with 60 μL of STM-LT2 culture supernatant, either simultaneously (orange bar) or 1 hour

apart (brown bar). LB medium (grey bar) and culture supernatant from STM-LT2 alone (green bar) were used as negative and positive controls, respectively. Data represented as geometric mean ± SEM, *n*=2. **(B)** Representative histograms from one volunteer treated as in (A) are shown. **(C)** Human monocyte-derived dendritic cells (MoDCs) were infected with violet-labelled (CellTracker™, Life Technologies) STM-D23580 or STM-LT2 at MOI of 10. At six hours post-infection, *Salmonella*-containing MoDCs (infected gate) were FACS sorted and co-cultured with autologous enriched $CD3^+$ T cells. Representative flow cytometry plots from one donor showing the percentage of TNF-α and/or IFN-γ producing MAIT cells. **(D)** MR1 overexpressing cells were incubated overnight in the presence of 50 µL of supernatants from bacterial cultures as indicated. 5-A-RU + MG and LB medium were included as positive and negative controls, respectively. Surface expression of MR1 was assessed by flow cytometry. Data represents geometric mean ± SEM, 1 biological replicate in duplicates. **(E)** Human MoDCs were co-cultured with non-autologous expanded human MAIT cells and infected with STM-D23580, STM-LT2 or *E. coli* at MOI of 3.5. Supernatants were harvested following 26 hours incubation and IL-12p70 was measured by ELISA, 1 biological replicate in triplicates.

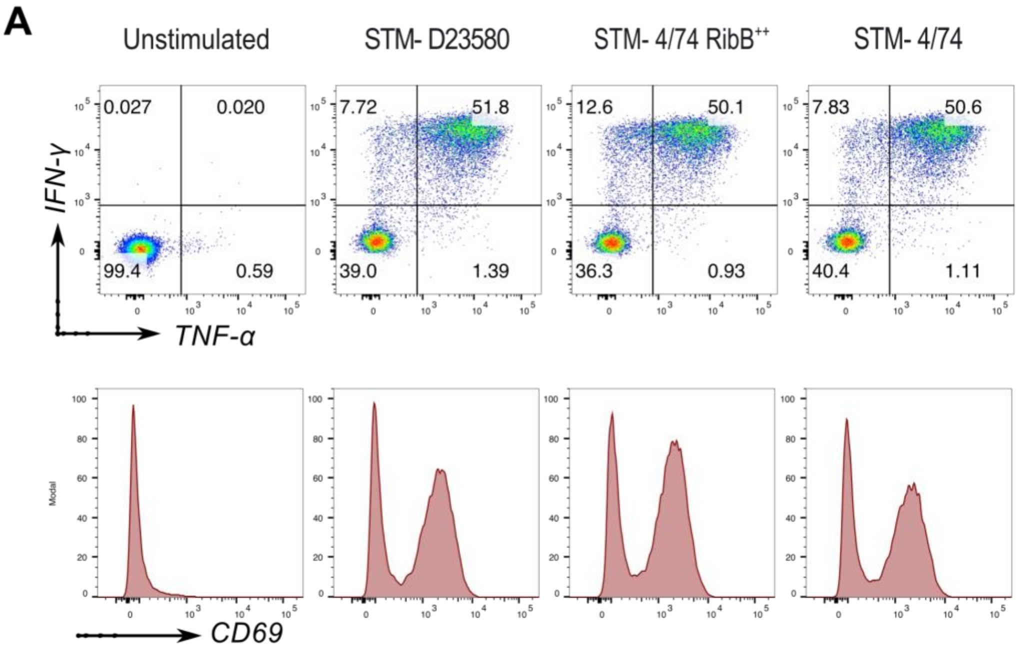

Figure S4. γδ T cell responses to STM-4/74 RibB++ are not different from STM-4/74 wild type.

PBMC were infected at MOI of 2 with STM-D23580, STM-4/74 or STM-4/74 RibB++. **(A)** Representative dot plots showing the percentage of TNF-α and/or IFN-γ producing γδ+ T cells. **(B)** Representative histograms for CD69 expression on γδ+ T cells from the same donor as in (A).

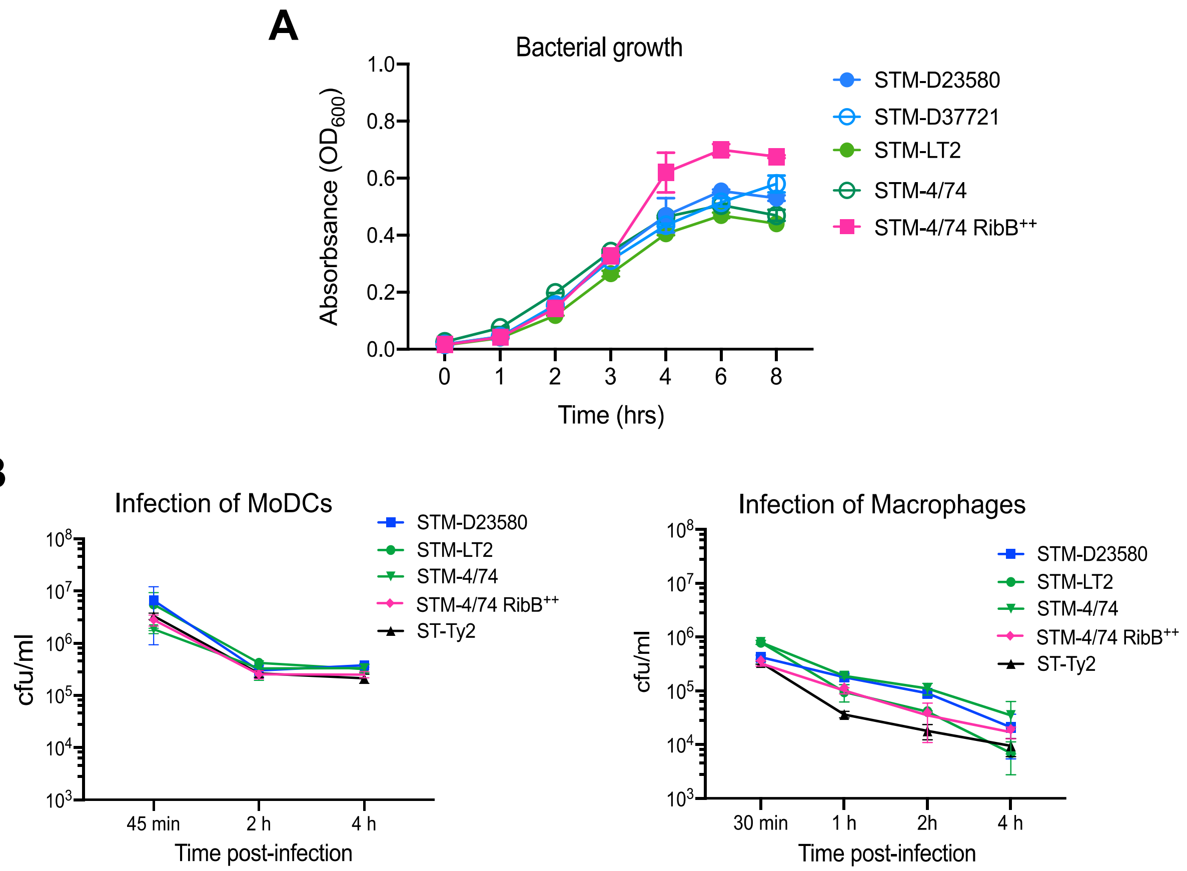

Figure S5. STM-4/74 RibB++ is a functional construct.

(A) Growth curves of the different *Salmonella* strains in LB lennox over an eight-hour period. Data is reported as absorbance (OD_{600}). Duplicates, mean ± SEM. **(B)** Human monocyte-derived dendritic cells (MoDCs, left panel) and human monocyte-derived macrophages (right panel) were infected at a MOI of 10 and 15, respectively. The number of intracellular bacteria at the indicated time points was obtained after cell lysis with saponin. Data is reported as colony forming units (cfu)/ml across timepoints. Two biological replicates, mean ± SEM.

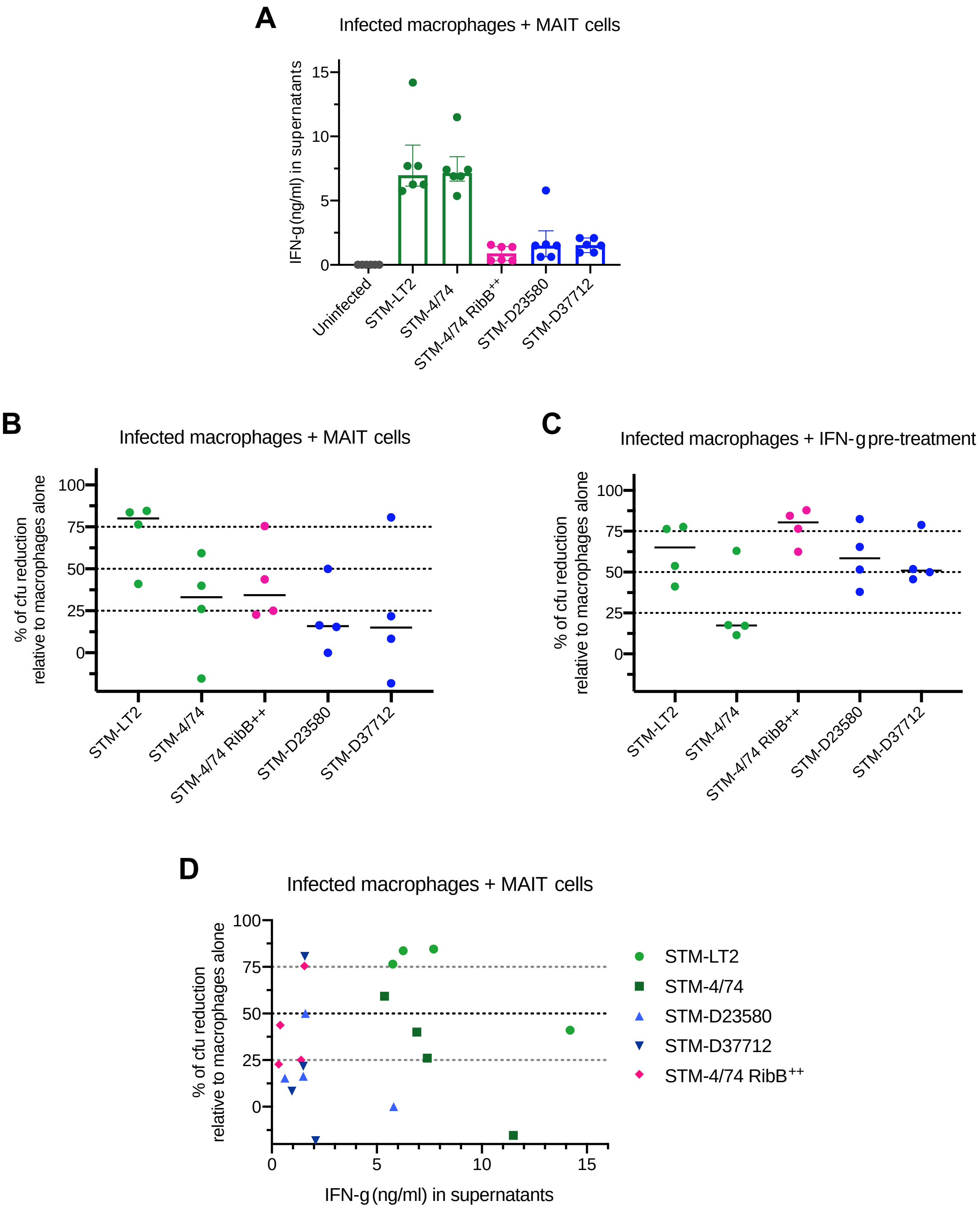

Figure S6. Reduced MAIT cell antibacterial activity against *Salmonella* sequence 313 lineage 2 infected macrophages.

Human monocyte-derived macrophages were infected with the different *Salmonella* strains at a MOI of 15, in the presence or absence of purified MAIT cells. **(A)** Supernatants were collected at 6 hours post-infection from the wells containing MAIT cells. IFN-γ levels were assessed by ELISA and reported in ng/ml. Six biological replicates for each group, median with IQR. **(B)** Infected macrophages were co-cultured with purified MAIT cells. The number

of intracellular bacteria from infected macrophages was obtained at 6 hours post-infection, after cell lysis with saponin. Data reported as % of reduction of cfu relative to the infected well without MAIT cells, four biological replicates for each group, line represents median. **(C)** Macrophages were pre-treated with commercial IFN-γ (100 ng/ml) from the night before infection. The number of intracellular bacteria from infected macrophages was obtained at 6 hours post-infection, after cell lysis with saponin. Data reported as % of reduction of cfu relative to the infected untreated well, four biological replicates for each group, line represents median. **(D)** Data from (A) and (B) is reported as a XY plot between the amount of IFN-γ in supernatants and the % of reduction of cfu relative to the infected macrophages without MAIT cells. Four biological replicates for each group.

Supplementary Table 1.

Resource	Source	Identifier
**Bacterial Strains**		
Salmonella Typhimurium 4/74 (ST19)	(Rankin & Taylor, 1966)	JH3676
Salmonella Typhimurium LT2 (ST19)	(McClelland et al., 2001)	ATCC 700220
Salmonella Typhimurium D25248 (ST313 lineage 1)	(Kingsley et al., 2009)	IC24T
Salmonella Typhimurium A082 (ST313 lineage 1)	(Kingsley et al., 2009)	NA
Salmonella Typhimurium A130 (ST313 lineage 1)	(Kingsley et al., 2009)	NA
Salmonella Typhimurium A357 (ST313 lineage 1)	(Kingsley et al., 2009)	NA
Salmonella Typhimurium A665 (ST313 lineage 1)	(Kingsley et al., 2009)	NA
Salmonella Typhimurium D23580 (ST313 lineage 2)	(Kingsley et al., 2009)	JH3621
Salmonella Typhimurium U60 (ST313 lineage 2)	(Ashton et al., 2017)	IC39S
Salmonella Typhimurium D36099 (ST313 lineage 2)	(Msefula et al., 2012)	NA
Salmonella Typhimurium D36448 (ST313 lineage 2)	(Msefula et al., 2012)	NA
Salmonella Typhimurium D36457 (ST313 lineage 2)	(Msefula et al., 2012)	NA
Salmonella Typhimurium D37712 (ST313 lineage 2)	(Msefula et al., 2012)	IC24O
Salmonella Typhimurium D36233 (ST313 lineage 2)	(Msefula et al., 2012)	NA
Salmonella Typhimurium D36435 (ST313 lineage 2)	(Msefula et al., 2012)	NA
Salmonella Typhimurium A50070 (ST313 lineage 2)	(Msefula et al., 2012)	NA
Salmonella Typhimurium U5 (UK-ST313 strain)	(Ashton et al., 2017)	IC25I
Salmonella Typhimurium U2 (UK-ST313 strain)	(Ashton et al., 2017)	IC25F

Salmonella Typhi Ty2	(Deng et al., 2003)	ATCC 700931
Salmonella Typhi Quailes	(Waddington et al., 2014)	NA
Salmonella Paratyphi NVGH308	(Dobinson et al., 2017)	NA
E. coli DH5α	ThermoFisher	NA
E. coli TOP10 (*mcrA D(mrr-hsdRMS-mcrBC)* ϕ8 *0lac*ZDM15 *ΔlacX74 deoR recA1 araD139* Δ*(ara-leu)7697galU galK rpsL endA1 nupG*)	Invitrogen	JH4317
E. coli TOP10 pP$_L$-*ribB*	This study	JH4318
S. Typhimurium 4/74 pP$_L$-*ribB*	This study	JH4319

NA: not applicable

Supplementary Table 2.

**Plasmids**	**Source**	**Identifier**
ColE1 control plasmid, based on pZE12-luc, P_{LlacO} promoter transcribes a ~50 nt nonsense transcript (*rrnB* terminator)	(Sittka, Pfeiffer, Tedin, & Vogel, 2007)	pJV300
pJV300 (pP$_L$) plasmid carrying the coding region of the *ribB* gene	This study	pP$_L$-*ribB*
**Oligonucleotides**		
GTGAGCGGATAACAAGATACTGAGCACCTGGTAACCATAATATTAATGAGG	This study	*ribB*_FW
GCCTTTCGTTTTATTTGATGCCTCTAGAATCAGCTGGCTTTGCGCTCATGCG	This study	*ribB*_RV
GTGCCACCTGACGTCTAAGA	This study	pPL_Seq_FW
ATACCGCTCGCCGCAGCCG	This study	pPL_Seq_RV

Supplementary References

Ashton, P. M., Owen, S. V, Kaindama, L., Rowe, W. P. M., Lane, C. R., Larkin, L., … Dallman, T. J. (2017). Public health surveillance in the UK revolutionises our understanding of the invasive Salmonella Typhimurium epidemic in Africa. *Genome Medicine*, *9*(1), 92. https://doi.org/10.1186/s13073-017-0480-7

Deng, W., Liou, S.-R., Plunkett, G., Mayhew, G. F., Rose, D. J., Burland, V., … Blattner, F. R. (2003). Comparative genomics of Salmonella enterica serovar Typhi strains Ty2 and CT18. *Journal of Bacteriology*, *185*(7), 2330–2337. https://doi.org/10.1128/JB.185.7.2330-2337.2003

Dobinson, H. C., Gibani, M. M., Jones, C., Thomaides-Brears, H. B., Voysey, M., Darton, T. C., … Pollard, A. J. (2017). Evaluation of the Clinical and Microbiological Response to Salmonella Paratyphi A Infection in the First Paratyphoid Human Challenge Model. *Clinical Infectious Diseases : An Official Publication of the Infectious Diseases Society of America*, *64*(8), 1066–1073. https://doi.org/10.1093/cid/cix042

Kingsley, R. A., Msefula, C. L., Thomson, N. R., Kariuki, S., Holt, K. E., Gordon, M. A., … Dougan, G. (2009). Epidemic multiple drug resistant Salmonella Typhimurium causing invasive disease in sub-Saharan Africa have a distinct genotype. *Genome Research*, *19*(12), 2279–2287. https://doi.org/10.1101/gr.091017.109

McClelland, M., Sanderson, K. E., Spieth, J., Clifton, S. W., Latreille, P., Courtney, L., … Wilson, R. K. (2001). Complete genome sequence of Salmonella enterica serovar Typhimurium LT2. *Nature*, *413*(6858), 852–856. https://doi.org/10.1038/35101614

Msefula, C. L., Kingsley, R. A., Gordon, M. A., Molyneux, E., Molyneux, M. E., MacLennan, C. A., … Heyderman, R. S. (2012). Genotypic homogeneity of multidrug resistant S. Typhimurium infecting distinct adult and childhood susceptibility groups in Blantyre, Malawi. *PloS One*, *7*(7), e42085. https://doi.org/10.1371/journal.pone.0042085

Rankin, J. D., & Taylor, R. J. (1966). The estimation of doses of Salmonella typhimurium suitable for the experimental production of disease in calves. *The Veterinary Record*, *78*(21), 706–707. Retrieved from http://www.ncbi.nlm.nih.gov/pubmed/5336163

Sittka, A., Pfeiffer, V., Tedin, K., & Vogel, J. (2007). The RNA chaperone Hfq is essential for the virulence of Salmonella typhimurium. *Molecular Microbiology*, *63*(1), 193–217. https://doi.org/10.1111/j.1365-2958.2006.05489.x

Waddington, C. S., Darton, T. C., Jones, C., Haworth, K., Peters, A., John, T., … Pollard, A. J. (2014). An outpatient, ambulant-design, controlled human infection model using escalating doses of salmonella typhi challenge delivered in sodium bicarbonate solution. *Clinical Infectious Diseases*, *58*(9), 1230–1240. https://doi.org/10.1093/cid/ciu078
